# A stochastic and dynamical view of pluripotency in mouse embryonic stem cells

**DOI:** 10.1371/journal.pcbi.1006000

**Published:** 2018-02-16

**Authors:** Yen Ting Lin, Peter G. Hufton, Esther J. Lee, Davit A. Potoyan

**Affiliations:** 1 Theoretical Division and Center for Nonlinear Studies, Los Alamos National Laboratory, Los Alamos, New Mexico, United States of America; 2 School of Physics and Astronomy, The University of Manchester, Manchester, United Kingdom; 3 Department of Bioengineering, Rice University, Houston, Texas, United States of America; 4 Department of Chemistry, Iowa State University, Ames, Iowa, United States of America; University of California Irvine, UNITED STATES

## Abstract

Pluripotent embryonic stem cells are of paramount importance for biomedical sciences because of their innate ability for self-renewal and differentiation into all major cell lines. The fateful decision to exit or remain in the pluripotent state is regulated by complex genetic regulatory networks. The rapid growth of single-cell sequencing data has greatly stimulated applications of statistical and machine learning methods for inferring topologies of pluripotency regulating genetic networks. The inferred network topologies, however, often only encode Boolean information while remaining silent about the roles of dynamics and molecular stochasticity inherent in gene expression. Herein we develop a framework for systematically extending Boolean-level network topologies into higher resolution models of networks which explicitly account for the promoter architectures and gene state switching dynamics. We show the framework to be useful for disentangling the various contributions that gene switching, external signaling, and network topology make to the global heterogeneity and dynamics of transcription factor populations. We find the pluripotent state of the network to be a steady state which is robust to global variations of gene switching rates which we argue are a good proxy for epigenetic states of individual promoters. The temporal dynamics of exiting the pluripotent state, on the other hand, is significantly influenced by the rates of genetic switching which makes cells more responsive to changes in extracellular signals.

## Author summary

In the embryonic stage mammalian cells are pluripotent: they have not yet committed to any specific cell type. The commitment to a cell type is controlled by pluripotency networks, the bio-molecular inventory which is unsurprisingly complex, spanning a myriad of transcription factors, genes and epigenetic factors. Thanks to advances in high-throughput sequencing and related computational tools for data analysis, we are beginning to unravel basic topological features of pluripotency networks. Networks inferred from sequencing data are often cast in Boolean representations which specify the existence and nature of regulatory connections between pairs of biomolecules. While immensely useful, the Boolean networks remain silent about the stochasticity and dynamics of molecules that are indelible features of bio-molecular life inside cells. Understanding stochastic and dynamic features of pluripotency networks is crucial if one is to have a mechanistic understanding of cell fate determination rationalized in terms of fundamental physico-chemical processes. The computational framework proposed in this work offers a way of bridging the divide between Boolean networks and higher resolution views of networks in a predictive and quantitative manner. The usefulness of the framework is demonstrated by recapitulating a number of experimental trends and creating new insights about the stochastic and dynamical nature of pluripotency.

## Introduction

Embryonic stem cells derived from mammalian blastocysts are pluripotent: they show an indefinite capacity for self-renewal and the ability to differentiate into every cell type constituting an adult organism [1–3]. The development of healthy tissues hinges on the ability of these pluripotent stem cells to make critical decisions determining when and into which kind of cells to differentiate in response to the environment. It is therefore unsurprising that fates of embryonic cells are decided through sophisticated biological computations by a tightly-integrated regulatory network consisting of genetic, epigenetic and signaling layers [3].

The expression of genes is subject to intrinsic noise due to finite molecular copy numbers [4] and due to randomness in extracellular environment. Thus, while at the level of the organism development is often predictable, with a well-defined order of events, at the level of single cells fate determination is fundamentally stochastic [5, 6]. Indeed, many studies probing the transcription of pluripotency-regulating genes in single cells have found high variability in distributions of transcription factors and mRNA molecules [7–9].

Several hypotheses about the functional roles for the highly heterogeneous expression of pluripotency transcription factors (TFs) have been put forward. One idea is that stochastic excursions in the population levels of transcription factors help by steering cells towards distinct states [10, 11]. A different hypothesis claims transcriptional noise to be advantageous by facilitating the exploration of the state space of a gene network such that, at any instant, a sub-population of cells is optimally primed to be responsive to differentiation signals [12]. The heterogeneity of populations of pluripotent cells has also raised some concerns that pluripotency is ill-defined at a single-cell level [12] and instead should be viewed as a macroscopic state emerginging at the level of an ensemble of cells. A comprehensive physical picture of pluripotency at the single-cell level therefore remains unclear.

In this regard, the roles of modeling and computational approaches are seen as especially important for bridging the gap between our understanding of molecular dynamics of regulatory networks and phenotypic outcomes. A rapid growth in single-cell sequencing data has opened many avenues for carrying out statistical inferences of pluripotency regulating genetic networks [13–15]. In vitro studies of mouse embryonic stem cells (mESC) in different culture conditions, in particular, have become an ideal model system for computationally inferring gene networks and exploring mechanistic issues surrounding pluripotency and lineage commitment [15]. In a *tour de force* study of mESC by Dunn *et al.* [16], regulatory relationships between transcription factors were uncovered through analysis of pairwise correlations in gene expression. Using mean values of RNA-seq counts as constraints, a minimal network topology was derived showing a high degree of predictive power with respect to perturbations of the network, such as gene knockouts.

Network topologies in general, however, remain silent about the roles of molecular noise and dynamics in stem cell differentiation governed by stochastic biochemical reactions. Furthermore, in order to validate that the inferred network reflects true microscopic reality of cell and is not a result of overfitting, one has to ultimately test the results using mass-action-based kinetics which integrate relevant molecular factors. The key challenge lies in finding the adequate resolution for the network which is able to be predictive and does not pose insurmountable computational burden.

In the present work, we outline a framework for extending Boolean resolution networks—a commonly derived product from high-throughput experimental results—into stochastic and dynamical models with microscopic resolution of promoter architecture and individual gene-switching events. The computational scheme utilizes static Boolean information about the network topology and uses novel analytical model reduction to increase the computational efficiency, allowing for extensive searches in the space of microscopic reaction rates. This framework is successfully applied to the network topology inferred by Dunn *et al.* [16] in order to build a mass action based stochastic dynamic model which is capable of describing both the discrete states of all the genes and the populations of transcription factors. Starting from minimal assumptions about the rates of various reactions, we find a parameter regimes where a remarkable agreement with the experimental gene expression profiles [16] is achieved for all combinations of external signals. We show that average gene-expression levels in complex regulatory networks are not a unique function of gene-switching rates, which cautions against over-interpreting Boolean-level networks and suggests strategies of inference which utilize higher moments in distribution of transcription factors. Using single cell experiments which have probed expression of pluripotency factors [7, 8], we are able to argue that gene switching in pluripotent states happens primarily on the intermediate scale relative to the reactions of production and degradation (dilution). This regime better agrees with the diverse set of experiments available [7–9, 16] and provides explanation for the multimodality in distributions of transcription factors and burst like expression dynamics for some genes.

In the second half of the paper, armed with a predictive and physically motivated model of pluripotency network, we explore the dynamics of lineage commitment driven by withdrawal of various well documented signals (LIF, 2i) for maintaining the naïve state of pluripotency. We find a number of non-trivial consequences of molecular noise and gene-switching dynamics. Taking gene switching rates as a proxy for epigenetic states of individual promoters we show that global variations of gene switching rates (mimicking global remodeling of epigenetic marks) yields a significant leverage over stability, sensitivity and exiting dynamics of pluripotent states In particular we show that intermediate gene-switching regime generates higher sensitivity for the network when responding to external signals.

## Results

### Framework for deriving microscopic resolution networks from experimentally inferred Boolean network topologies

In this Section we outline the steps for constructing higher-resolution regulatory networks with explicit promoter architecture, gene states, and transcription factor copy numbers, starting from experimentally-inferred Boolean network topologies. To illustrate the potential of our modeling framework we have chosen the most comprehensive Boolean network to date which describes the regulation of pluripotency factors of mouse embryonic stem cells [16]. In the next subsection we describe how Boolean logic is converted into the molecular logic of promoter states via a set of simplifying assumptions. Once promoter logic is defined, in the second subsections we go on to define the resolution of protein to promoter interactions.

These assumptions lead to a set of chemical reactions describing transitions between gene states. In the third subsection we introduce the rules defining the production and degradation of transcription factors. In the fourth subsection we give an overview of the multi-scale simulation techniques appropriate for the simulation of stochastic switching of genetic states with a single-molecule-level resolution. In particular we contrast the rigorous yet computationally inefficient individual-based model (IB) with a more efficient piecewise-deterministic Markov Process (PDMP) which we adopt and extensively validate in the present work.

### Extending the Boolean logic to molecular logic

**Fig 1:**
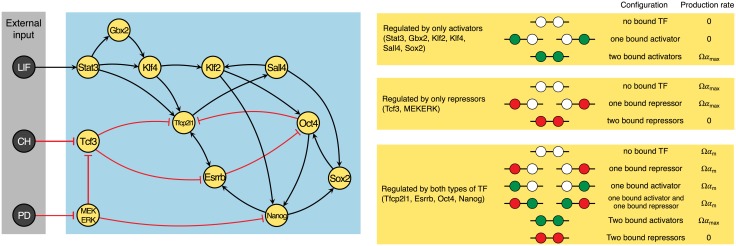
Network topology and molecular logic. The left panel shows a schematic diagram of the network topology, reproduced from Dunn *et al.* [16]. Each node corresponds to a given gene and their placement from left to right is chosen to indicate a trend of downstreamness from three external inputs. In our mechanistic model, each gene produces a unique transcription factor at a rate which depending on the binding state of its promoter site. These transcription factors then go on to bind and activate (black arrow) or repress (red bar) other genes. The three nodes on left correspond to extra-cellular signals, which are either absent or present. The right panel shows our assumed molecular logic of transcriptional regulation when there are *N* = 2 promoter sites per gene. Each circle is a binding site: it can be either empty (white), bound by an activator (green), or bound by a repressor (red). The right panel lists possible combinations of the promoter sites. Depending on the configuration of the promoter site, transcription factors are produced with rates 0, Ω*α*_*m*_, and Ω*α*_max_, modeling the effects of cooperative binding.

The Boolean model of Dunn *et al.* specifies twelve genes in the network. We use *G*_*i*_ to denote a gene and *P*_*i*_ to denote the corresponding functional TFs (*i* ∈ {1, 2, …, 12}) in the following framework. The network topology (Fig 1) contains static information about the types of interactions between pairs of genes. The interactions are classified as being either repressing or activating. To study the dynamics of complex genetic networks, one has to extend the Boolean-level description to account for the molecular logic of gene regulation. This molecular logic specifies the precise relation between the binding of transcription factors to the promoters and the regulatory outcome in terms of gene activation or repression. In the case where the same sites can be bound to different transcription factors, the combinatorial nature of regulation can give rise to ambiguity in molecular logic. Indeed, even on the level of Boolean networks, the logic is ambiguous and many possible truth tables have to be enumerated in order to select an appropriate picture for the dependence of a gene on its regulating signals. Ideally, such ambiguities should be resolved by directly inferring regulatory logic. Alternatively, one could simulate different combinatorial possibilities until sufficient agreement with experiments is reached.

In our case, we assume a set of rules which applies to every gene in the network. This is a simplifying assumption, the justification for which is obtained *a posteriori*; networks with optimized parameters yield gene expression patterns consistent with the experiments. The fact that these simplifying assumptions work surprisingly well implies a dominance of the global network topology over the local details of molecular regulatory logic of gene promoters.

For each gene, we assume a fixed number *N* of promoter sites. The genes which are only regulated by activators are always “OFF” unless *N* promoter sites are bound to activators. Similarly, the genes which are only regulated by repressors are always “ON” unless *N* promoter sites are bound to repressors. For those genes which are regulated by both activators and repressors, their activity is up-regulated to “ON” (resp. down-regulated to “OFF”) only when *N* promoter sites are bound to activators (resp. repressors), otherwise they have a “MEDIUM” activity. These rules are illustrated quantitatively in the right panel of Fig 1.

### Binding and unbinding of transcription factors to promoter sites

From the topology, we know that each gene is regulated (activated or repressed) by a subset of TFs; we denote the subset of the TFs which regulates gene *i* by *S*_*i*_ ∈ {*P*_1_, *P*_2_, …, *P*_12_}. For example, the subset of the TFs which regulates the core gene Nanog is the set of transcription factors Klf2,Sox2, MEKERK (see Fig 1). The binding and unbinding of the transcription factors to the promoter sites of gene *i* can be summarized by the following binding and unbinding reactions:

$$\begin{array}{ccc} G_{i}+P_{j}\overset{Nk_{\text{on}}\Omega^{-1}}{\underset{k_{\text{off}}}{⇌}}G_{i}P_{j} & \;\text{for}\;P_{j}\in S_{i}, & \left(\text{first}\;\text{TF}\right)\\ \vdots & & \\ G_{i}P_{j}\ldots \;P_{k}+P_{l}\overset{k_{\text{on}}\Omega^{-1}}{\underset{Nk_{\text{off}}}{⇌}}G_{i}P_{j}\ldots \;P_{k}P_{l} & \;\text{for}\;P_{l}\in S_{i}, & \left(\text{first}\;\text{TF}\right) \end{array}$$ (1)

where the rates are written per the reactants, i.e., a given *P*_*j*_ binds to a given unbound promoter *G*_*i*_ with rate *Nk*_on_Ω^−1^ etc. The “system size” parameter Ω has been introduced which sets the scale for a typical number of TFs in the system [4, 17] without loss of generality. The scaling of these switching reactions is chosen such that the time scale of gene switching is independent of Ω [18]. Notice that we have adopted the parallel binding mechanism [19] which assumes the transcription factors bind to any of the unbound promoter sites independently with a rate constant *k*_on_.

### Production and degradation of transcription factors

Each gene *G*_*i*_ “produces” its own transcription factor *P*_*i*_ with a rate which depends on the promoter state of *G*_*i*_. We use a one-step model of transcription [20] whereby transcription factors are produced via a unimolecular step. This approximation coarse grains several sequential intermediate reactions (polymerase recruitment, mRNA production, RNA splicing, etc.) into one effective step [21, 22]. This approximation has become popular in models of protein production [18, 23, 24]. All of the transcription factors are assumed to have finite lifetimes set by the rate of degradation, thus ensuring the existence of stable steady states with a finite number of molecules. The absolute time is set to be ∼ 1 hr which is consistent with experimental measurements reported on the lifetimes of pluripotency transcription factors [25–28].

The reactions are

$$\begin{array}{c} \begin{array}{ccc} G_{i} & \overset{\Omega \alpha }{\underset{\;}{\to }}G_{i}+P_{i}, & \text{(TF}\;\text{production)}\\ P_{i} & \overset{\gamma }{\underset{\;}{\to }}⌀. & \left(\text{TF}\;\text{degradation}\right) \end{array} \end{array}$$ (2)

Here, the rate constant of the production of TFs (Ω*α*) depends on the promoter’s configuration and is determined by the molecular logic defined previously. We further assume the production rate when the gene is “ON”, “MEDIUM”, and “OFF” to be Ω*α*_max_, Ω*α*_*m*_, and 0 (see Fig 1). These rates are taken to be identical among all the genes.

We note that one can adopt a view with a higher resolution of the network depending on the available experimental data and the nature of the question posed. The computational framework can readily incorporate in an explicit manner more steps, for instance to model the effects of cell cycle regulation, different epigenetic states, and binding of non-coding RNAs. Herein we consider the most simplified dynamical model which describes only the promoter configurations and the population dynamics of the transcription factors. This model aims to use the optimal resolution for capturing trends in gene expression while remaining feasible for efficient stochastic simulations. After converting the Boolean topology into a higher resolution network of biochemical reactions, our next goal is to exhaustively sample a vast space of parameter space in order to identify the optimal parameter regimes with which the model best reproduces the experimental results.

### Multi-scale simulation of complex genetic networks

Biochemical reactions in gene networks are of a fundamentally probabilistic nature; this demands a stochastic description of the kinetics. The conventional mean field mass-action-based kinetics only describe the dynamical behavior in the thermodynamic limit, and ignore the stochastic effects which arise from the discreteness of the system. In our model, the system is highly discrete, as there is only a single copy of each gene, and their discrete promoter states dictate the dynamics of the transcription factors. The most rigorous way to simulate such a reaction system is by numerically solving the chemical master equation which accounts for all possible states of the network down to the level of single molecules [4, 20]. In high dimensions, i.e., when the number of species is large, this approach is not computationally efficient. Instead, kinetic Monte Carlo algorithms are the most straightforward way to generate sample paths of the stochastic process. We refer to the stochastic process modeling the reactions down to the single-molecule level with the well-mixed assumption as the individual-based model (IB).

The state of our individual-based model is characterized by the state of each gene’s promoter sites—how many sites are bound to specific kinds of transcription factors—and the integer populations of the transcription factors. The rates at which the process stochastically transitions from one state to another are specified by the reactions Eqs (1) and (2). Fully individual-based models, however, still suffer from a steep scaling of computational time with the number of discrete system states. This fact renders them inefficient for simulating large gene networks, especially when it comes to scanning or exploring the parameter space.

A wide range of approximate schemes have been employed to simulate complex gene regulatory networks [29–31]. Most conventional approximations so far have been the size-expansion methods [32, 33] which are known for being problematic when the molecular noise induced by discrete genetic switching becomes non-negligible [18, 34, 35]. On the other hand, for embryonic stem cell networks it is essential to account for the stochastic nature of genetic switching events which give rise to multimodality in the probability distributions of transcription factors populations. These local maxima in the multidimensional probability distributions of a stochastic genetic network correspond to metastable states and are also known as local attractors which corresponding to distinct promoter configurations and hence likely also to distinct phenotypic states [36–38]. Theoretically a network with *M* independently functioning genes can generate up to ∼ 2^*M*^ distinct phenotypic states [39].

Thus, even if populations of all the other species are present in large quantities, the stochastic fluctuations caused by the genetic switches (due to stochastic binding-unbinding events of the transcriptional factors to a discrete number of promoter sites) between ON, MEDIUM, and OFF states cannot be ignored, unless the switching is operating in the extremely fast limit compared to any other reactions, known as the “adiabatic regime” [35, 39, 40]. In the other cases—the non-adiabatic regime—gene switching can completely dominate the dynamics in the network [35, 41]. Eukaryotic gene regulatory networks are often found in the intermediate regime where gene-switching events are dynamically interwoven with the rest of the reactions in the network and cannot be ignored [5, 42, 43]. Single-cell studies of mESC in particular have shown bursty behavior in gene expression with sudden jumps in the levels of proteins resulting in multi-modal distributions of core transcription factors [7].

The sheer volume and complexity of information that is emerging from experiments on embryonic stem cells have motivated the application of a wide variety of modeling strategies for confronting the observed patterns of gene expression in ESCs [44, 45]. Some of the computational techniques used so far include Boolean networks [16, 46–48], Hopfield neural networks [49–51], systems of coupled ordinary [52, 53] and stochastic differential equations [10, 54, 55] with Hill coefficients, agent-based models [56], individual-based models [43, 57, 58] and small noise approximations to individual-based models [59–61].

Many of the early computational models of embryonic stem cells focused on small fragments of pluripotency networks, typically involving bistable switches [10, 52]. These early pioneering studies yielded many insights on stochastic decision making in regards to fate determination and self-renewal [44]. Relatively few studies have also looked at larger portions of regulatory networks while including a stochastic treatment of genetic switching dynamics by either carrying out individual-based simulations [43, 57] or small noise approximations [59–62].

Because of the computationally demanding nature of individual-based models and the restricted validity of small noise approximations to the near-adiabatic gene switching, the full range of stochastic and dynamical regimes displayed by different gene switching time-scales has remained unexplored. Additionally with very few exceptions [43], the kinetic parameters in many of the previous models have been not thoroughly explored or informed by data and have had to be selected from physical intuition alone. These limitations have now been overcome in the present work. Thanks to a series of recent developments in modeling of gene expression dynamics [18, 23, 24, 35, 63–65], a novel computational framework utilizing piecewise-deterministic Markov processes (PDMPs) [66] has emerged as a rigorous approximation to the fully individual-based model.

The idea behind PDMP is simple: reactions with large number of molecules are evolved deterministically, while reactions with smaller numbers of molecules are propagated as discrete, random switching events. This approach treats discrete genetic switching events exactly, while assuming noise due to the finite nature of populations of transcription factors to be small in comparison. As shown in the subsequent sections, the assumptions underlying the PDMP approach turn out to be sound as we go on to obtain a nearly perfect quantitative agreement with full blown kinetic Monte Carlo schemes even for the case of the complex networks of mESC operating in the intermediate gene-switching regime. The mathematical details of the PDMP are given in the next subsection.

The PDMP simulations carried out in the present study show nearly $O(10^{3})$ fold faster generating stochastic trajectories compared to conventional individual-based kinetic Monte Carlo techniques (the algorithm for generating PDMP sample paths is provided in the Method section). This rigorous and rapid sampling of gene-switching events has not only allowed us to investigate the stochastic dynamics of the regulatory network at a longer time scale compared to conventional kinetic Monte Carlo methods, but also enabled us to explore a vast parameter space efficiently. We have used the obtained information to derive microscopic resolution models of ESCs. The mathematical details of the PDMP described in greater detail can be found in the next few subsections.

### In the pluripotent state the mean levels of gene expression are robust with respect to changes of gene state switching rates

After converting the Boolean topology to a higher resolution genetic network we use a computationally efficient stochastic dynamics method (PDMP in the present case) to explore the kinetic parameter regimes. This is done in order to: i) Identify all the unique steady states ii) Obtain a set of rate coefficients that best reflects the experimental constrains iii) Study the response of the network to gene switching dynamics the rate of which is taken as a proxy for epigenetic states. The experimental data used for constraining rate coefficients are the binarized gene expression levels of pluripotency transcription factors under well-defined culture conditions consisting of different combinations of leukemia inhibitory factor (LIF), glycogen synthase kinase 3 (CH), and mitogen-activated protein kinase (PD). Identical culture conditions were used by Dunn *et al.* [16] to infer the original Boolean topology.

The optimization of rate coefficients is done by defining a uniform threshold *η* among all the TFs and with different external conditions; TFs whose population densities are higher than *η* are classified as expressed, while those below the threshold are not expressed. Then, we employ the Hamming distance (the number of discrepancies between the simulated and experimental profiles) as a cost-function for the optimization. The Hamming distance is minimized through multiple rounds of simulations where we vary the uniform threshold *η* and four free and non-dimensional model parameters (see Method section): the number of promoter sites per gene *N*, the rate constant of TFs binding to the promoter *k*_on_, rate constant for a bound TF to dissociate from the promoter *k*_off_, and the production rate *α*_*m*_ when the gene is in the “MEDIUM” state.

**Fig 2:**
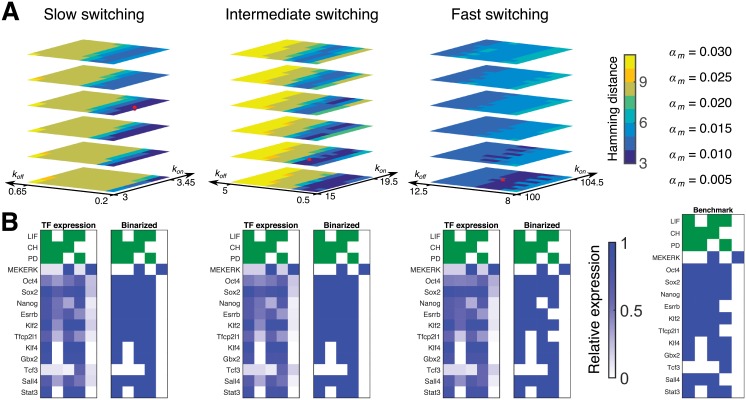
PDMP stochastic simulations identify three genetic switching regimes that are consistent with experimental data. When switching is slow, intermediate, or fast we find certain parameters which closely match the experimental results obtained by Dunn *et al.* [16]. The consistency of our model with experimental results is measured using a Hamming distance—a measure where one counts the number of discrepancies between the binary expression of each TF for both cases. (A) Shown are the identified regions in parameter regimes that minimize Hamming distance. There are three free parameters: the binding rate *k*_on_, unbinding rate *k*_off_, and basal transcription rate *α*_*m*_. For slow switching, the parameters are *k*_on_ = 3.2, *k*_off_ = 0.2, *α*_*m*_ = 0.02; for intermediate switching, *k*_on_ = 16, *k*_off_ = 1.5, *α*_*m*_ = 0.01; for fast switching, *k*_on_ = 102, *k*_off_ = 10, *α*_*m*_ = 0.005. The selected parameter sets are presented as red dots in the landscapes in the upper panel, hereby referred to as *the* slow, fast and intermediate parameter regimes. (B) Comparison of computed and discretized gene expression profiles with those of the experiments (Benchmark panel) [16].

Through this procedure, we find that the binary expression patterns can be closely reproduced (minimal Hamming distance = 3) by the model when the number of the promoter sties *N* ≥ 2. We remark that, given the small number of free parameters, this is a very small amount of deviation from the experimental results; the remaining deviations are likely to have been caused by the simplifications to the logic and dynamics in the construction of our individual-based model. The qualitative features for *N* = 2, 3, … 5 cases are similar (see S2–S7 Figs in the Supporting Information). We chose to present the data for the simplest case *N* = 2 in the manuscript. We note that various other forms of cooperativity, including cases when bound TF at the promoter recruits other TFs (either of its own kind or other type), can be readily incorporated into the current computational framework by changing the association rates *k*_on_ as promoter-state dependent. For simplicity, we only illustrate the most basic form of cooperativity in this pilot paper. We find for any given *N* there exists a “valley” in the remaining model parameter space *k*_on_, *k*_off_, *α*_*m*_, as illustrated in Fig 2(A). The inferred parameter *α*_*m*_ is consistently low (≲ 0.02), meaning that the “MEDIUM” production rate is almost zero. This implies that the negative regulation of those genes which are regulated both by activators and repressors (Tfcp2l1, Esrrb, Nanog, and Oct4) in the model are technically fulfilled by inhibition (i.e., regulating TFs by blocking the promoter sites), instead of actually down-regulating the production activity. In addition, the valley suggests a relationship between *k*_on_ and *k*_off_, where *k*_on_ ≈ 10*k*_off_. This implies a certain asymmetric time scale between the binding and unbinding processes.

We chose three parameter sets in this valley of cost function, corresponding to three distinct dynamical regimes of binding and unbinding reactions between the TFs and the promoter sites: slow (*k*_on_ = 3.2, *k*_off_ = 0.2, *α*_*m*_ = 0.02), intermediate (*k*_on_ = 16, *k*_off_ = 1.5, *α*_*m*_ = 0.01), and fast (*k*_on_ = 102, *k*_off_ = 10, *α*_*m*_ = 0.005) compared to the time scale of the TF dynamics. We remark that as we non-dimensionalize the model using the protein degradation rate *γ* (see Method section), the time scale of the binding and unbinding are fast, intermediate, or slow *relative* relative to the time scale of the protein dynamics, also set by *γ*. See Eq 3. All of the parameter sets successfully reproduce the experimental gene expression patterns (Fig 2) corresponding to pluripotent and lineage committed cells.

The fact that the rate parameters of the network occupy finite regions and cover different regimes suggests that distinct gene expression profiles can tolerate fluctuations in reaction rates. Such rate fluctuations, reflecting the effect of extrinsic noise, are inevitable in dynamic cellular environments of embryonic cells which experience frequent epigenetic and extracellular perturbations [15, 26, 67]. In a way, changes in gene-switching rates can be seen as a proxy for how global epigenetic changes govern the rates of transcription factor binding to target genomic regions.

**Fig 3:**
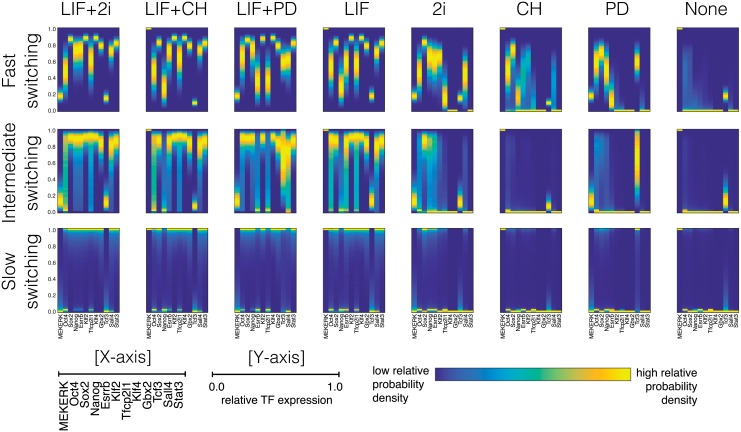
Gene expression profiles of pluripotency factors predicted by PDMP simulations. Each column corresponds to different external inputs, and each row corresponds to regimes of slow, intermediate and fast gene switching. More than 10^5^ sample paths were used for generating each condition.

From the methodology point of view, the absence of a unique regime of rates implies the following: inferring networks using only mean levels of gene expression (as is done for Boolean networks) may lead to the loss of valuable information contained in higher moments of distribution. Thus new approaches of inference need to be developed in order to account for broad distributions of transcription factors. For this reason, we look beyond the comparisons of mean expression levels and turn to comparing stationary distributions of transcription factors observed in experiments with the computationally generated distributions in three chosen parameter regimes identified in Fig 2. We generate the stationary distributions of the TF concentrations in the model, with eight different environmental conditions, in the specified three parameter sets in Fig 3. We observe qualitatively that in the “fast” parameter set the distribution of each TF is unimodal, and in the “slow” parameter set, the distribution of each TF is peaked near “0” and “1” in non-dimensional units. In the intermediate regime, on the other hand, with some environmental conditions, broader distributions of the gene expression are observed.

The pluripotent state of mESCs has been the subject of intense investigations by nucleic acid-based single-cell techniques such as RNA-seq, sm-FISH, qPCR and there is now extensive data on the steady-state distributions of RNA and transcription factors maintained under pluripotency-favoring culture conditions [7, 8, 68]. These experiments have revealed a heterogeneous nature of gene expression with many core pluripotency factors, such as Nanog and Esrrb, having long-tailed or bimodal distributions (see for example Fig. 5a in Ref. [7]). Additionally, the single-cell stochastic trajectories of TFs have shown sharp, bursty transitions implying infrequent genetic switching events [7]. Qualitatively, these experimental observations are more consistent with the intermediate regime of genetic switching as seen from Fig 3, as in the case of slow switching nearly all of the transcription factors express bimodality and in the case of fast switching the expressions of all the factors are narrow and unimodal.

In Fig 3, consistent with experiments, we also find that under LIF gene expression is more heterogeneous than under 2i, but also that the overall levels of expression are higher [7, 8]. In all three regimes of genetic switching supporting pluripotency (LIF+2i,LIF+PD, LIF+CH, LIF and 2i), core transcriptions factors such as Nanog, Oct4, Sox2 are highly expressed. The same factors are also repressed in conditions favoring differentiation (CH, PD and none) irrespective of gene-switching regime. This shows that the pluripotent and differentiated states, as determined by the pattern of gene expression, are hardwired in the architecture of the genetic network independent of genetic switching rates. Nevertheless, as we will show in the next section, the routes and dynamics of lineage commitment from pluripotent states strongly depend on the level of molecular noise generated in different gene-switching regimes.

**Fig 4:**
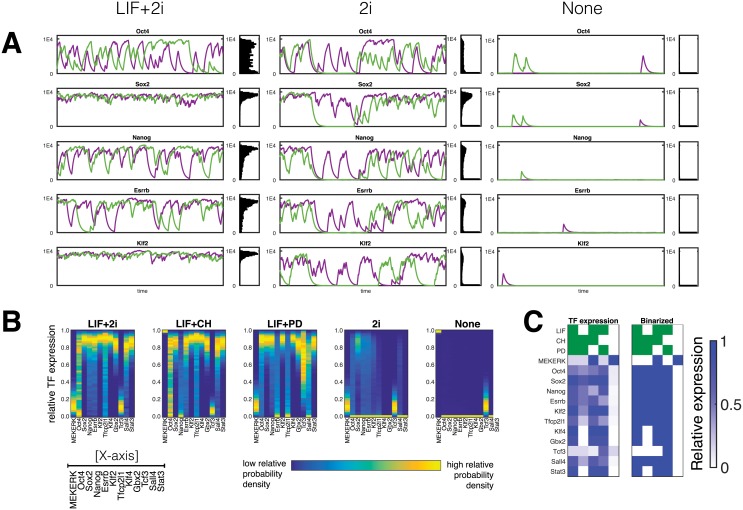
Gene expression profiles of pluripotency factors predicted by individual-based simulations. The intermediate switching regime is chosen to be presented as it is the regime which best captures the experimentally measured distributions [7, 8]. (A) Shown are two representative trajectories and full distributions of select few transcription factors under three different conditions generated by individual-based simulations. Typical lifetime for pluripotency transcription factors [25–28] (∼ 1 hr) is used for setting the absolute time-scale of simulations (B) Gene expression profile showing the near quantitative agreement with results of PDMP simulations shown in Fig 3. (C) Gene expression profile of individual-based model, for comparison with the PDMP and experimental benchmark shown in Fig 2.

Although the PDMP approach accurately captures the effects of genetic switching, it assumes demographic noise arising from finite populations to be negligible. To test the validity of this assumption and assess the contribution of different sources of noise in establishing the steady-state distribution of the pluripotent state, we carry out individual-based simulations for the intermediate switching regime of the network, Fig 4. In the individual-based model all reactions are treated stochastically thereby accounting for all of the sources of noise in the system. The resulting gene expression profiles follow closely those obtained by PDMP simulations, showing that the noise arising from stochastic switching events of promoter configuration accounts for the significant part of overall variability in the network. Trajectories of individual transcription factors show that indeed most of the variance in the molecular distributions are generated by genetic switching events which appear as abrupt stochastic jumps.

### Dynamics of lineage commitment is significantly affected by the dynamical changes of individual gene-switching rates

We next ask how the steady-state gene expression patterns displayed by the gene network respond to extracellular perturbations in the form of the initiation or termination of pluripotency signals. Both dual inhibitor 2i (PD+CH) and Leukemia factor LIF based signaling have been shown to provide a stable environment for maintaining pluripotency of stem cells in vitro [3, 16]. Conversely, withdrawal of either LIF or 2i leads to irreversible lineage commitment after a 24-hour period. Despite a similar ability to guard pluripotent cells against lineage commitment, these factors deploy different regulatory mechanisms reflected in distinct distributions of pluripotency factors. As a result, stem cell differentiation by withdrawal of different signals proceeds via different routes. To gain a mechanistic understanding of how the interplay of signaling, molecular noise and network architecture gives rise to the steady-state expression profiles, we study the dynamics of transitioning between the pluripotent and lineage committed states induced by rapid initiation and withdrawal of signaling conditions (LIF, CH, PD).

**Fig 5:**
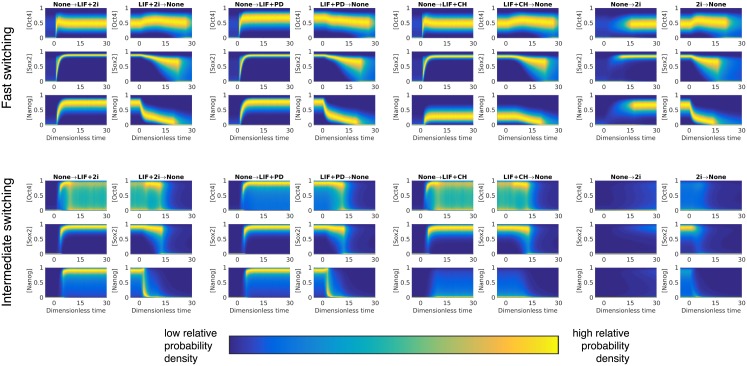
The dynamical behavior of the distributions of transcription factor densities for the intermediate and fast switching regimes. At time *t* = 0, the external inputs are changed. The plots show the evolution in probability density for a large ensemble (10^5^) of PDMP sample paths. The times and populations are respectively rescaled by 1/*γ* and *α*_max_/*γ* (see Method section). We assume the proteins are stable and so the their estimated half-life is of an order 8 hour [7]; in other words *γ* ∼ 1/8 hr^−1^, and the entire course of simulation (*t* ∈ (0, 30)) corresponds to physically 240 hours. A validation of the PDMP prediction by individual-based simulations can be found in S8 Fig in the Supporting Information.

The temporal evolution of distributions of the TFs exiting (LIF/2i withdrawal) and entering (LIF/2i immersion) pluripotent states reveals rich, dynamical signatures of these transitions (Fig 5). To reveal the role of stochasticity in these transitions, we compare the intermediate regime—which is dominated by genetic switching—to the fast regime—in which transitions are largely governed by the network topology and the fluctuation of promoter configuration is almost-completely ignored. The irreversible nature of transitions manifests clearly in different routes exiting and entering the pluripotent state (transitions to and from the None state in Fig 5).

**Fig 6:**
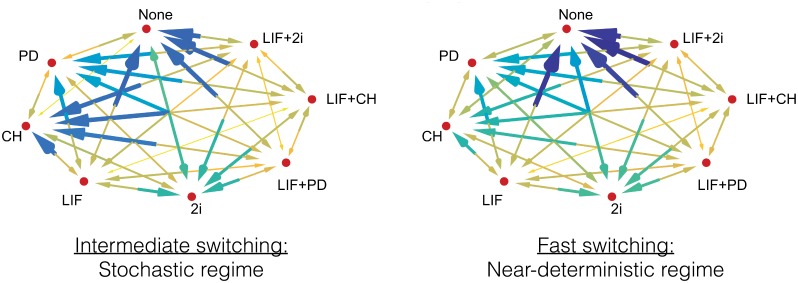
Transition times between the stationary distributions of different external conditions. A larger, darker arrow indicates that a given transition takes a longer time to converge to its stationary state. This time scale is measured by simulating a large ensemble (10^5^) of PDMP sample paths to provide a simulated probability density and finding the Jensen–Shannon divergence [69, 70] between the instantaneous distribution of each TF and its final stationary distribution. The time for the each divergence to fall below a threshold (≔ 0.3) is recorded, and we choose the largest of these as a quantification of the transition time. The numerical values can be found in S1 and S2 Tables in the Supporting Information.

In the intermediate regime of genetic switching rates, expression noise greatly facilitates transitions out of a pluripotent state by making the network more responsive to changes in environmental signaling. In contrast, in the fast-switching regime, upon withdrawal of pluripotency signals the downstream regulation happens on a much slower time scale with some factors remaining virtually unresponsive to changes of signaling. This signaling enhancement in the intermediate regime reveals the importance of molecular noise in making pluripotent states more sensitive to environmental conditions. There are qualitatively different patterns of re-entrance into pluripotent states upon LIF vs 2i addition, with LIF being much more efficient at reversing pluripotency compared to the 2i. The different potentials of signaling cultures for pluripotency reversal has been established in experiments [3] which have shown that in the later stages of commitment only the LIF is able to reverse lineage-primed cells back to their naive pluripotent states. The exit and re-entrance from pluripotency upon withdrawal and addition of LIF shows complex signatures of hysteresis and bifurcations. This suggests that there can be multiple pathways of entering or exiting pluripotency. The transition times for all signaling-induced changes of the steady states of the network are visualized on a kinetic diagram, Fig 6. This figure shows an underlying structure to these transitions where conversion among pluripotent states takes place with lower “activation barriers” compared to transitions accompanying loss of pluripotency. In the intermediate switching regime there is a clear time scale separation between transitions which keep cells in pluripotent states and transitions out of pluripotent states. One may argue that such a time scale separation between signals inducing differentiation and pluripotency allows embryonic cells to execute developmental decisions more faithfully. In the fast-switching regime this clear time scale separation is partially lost where only the loss of all three signals is separated from the rest of the transitions.

Detailed analysis of individual distributions and trajectories of transcription factors can be very informative due to their high information content. It is, however, not immediately clear how changes in the expression of individual genes contribute to global changes corresponding to different phenotypic transitions. To reveal such global changes, we project stochastic trajectories of all transcription factors onto the first two eigenvectors obtained by principal component analysis (PCA) of the reference pluripotent steady state (LIF+2i). Most of the variance of transcription factors is well captured by the few principal components. The high-dimensional steady state of the cellular network can thus be conveniently projected onto a 2-dimensional subspace, allowing us to visualize the attractor states of the network as probability landscapes *π*(*PC*_1_, *PC*_2_) which are often masked by heterogeneous distributions.

**Fig 7:**
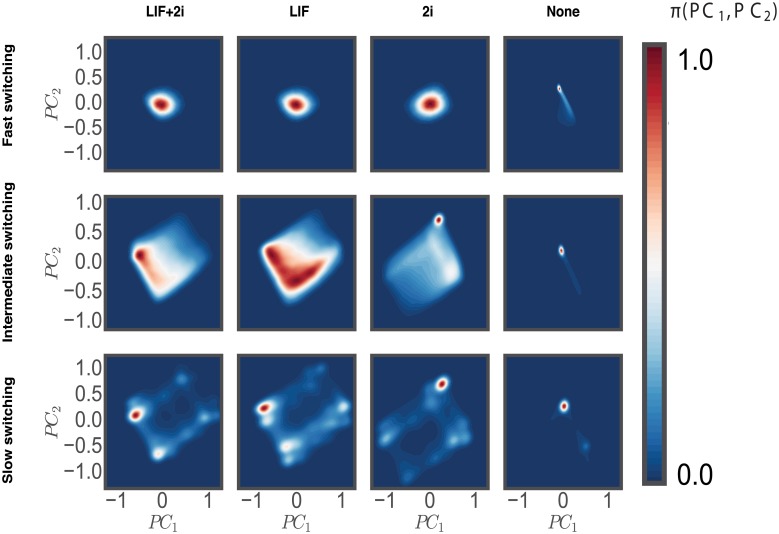
Mapping the cellular attractors of the genetic network under different switching and signaling conditions. We project PDMP-simulated gene expression distributions onto the first two principal components. The reference state for principal components was chosen to be the LIF+2i/intermediate switching.

Comparing these probability landscapes with different gene-switching regimes reveals the distinct roles played by gene switching-induced molecular noise and the deterministic network topology in guiding the transition out of the pluripotent states (Fig 7). The intermediate gene-switching regime, once again, appears to be the more viable regime underlying pluripotent states since the probability landscape shows up as a broad attractor with interconnected states. Going towards the limit of slow switching results in the fragmentation of the landscape into states separated by high barriers. This gene switching-induced remodeling of attractors shows the potential for regulation via global epigenetic changes which are purported to act via silencing or activating entire sets of genes at once [15]. Thus one may view gene-switching rates as a proxy for genome wide acetylation/methylation patterns which can dramatically alter the access of transcription factors to key target genomic sites. The sequential removal of pluripotency-inducing signals reveals a consistent change in the size of the attractor towards occupying smaller regions on the landscape. This argues for the physical state of the network corresponding to pluripotent states to be the one with maximal variance of regulatory transcription factors where lineage commitment is accompanied by their gradual constraining and repression. A similar idea which views pluripotency as a macrostate emerging from an ensemble of cells which try to maximizes the information entropy with respect to regulatory transcription factors has been postulated before [12, 71]. The analysis of the steady-state stochastic dynamics of the pluripotency network in this work appears in agreement with this view. Furthermore, we are able to suggest a microscopic origin of this entropic paradigm. By analyzing pairwise correlations among different transcription factors we find that signals like LIF/2i create greater independence between the expression of core transcription factors, leading them to explore a larger range of values. Hence, removal of these signals leads to more constrained and interdependent patterns of gene expression for the same transcription factors which greatly diminishes overall variance (see S1 Fig).

## Discussion

Experimental [16, 51, 72] and computational studies [54, 56, 73, 74] of embryonic stem cell networks have increasingly emphasized a systems-level perspective where pluripotency is a result of sophisticated biological computations done by tightly integrated set of genes, epigenetic and transcription factors [16, 75]. The rapid growth of gene-expression data on mESCs under many different *in vitro* conditions has enabled reliable inferences of the underlying topology of pluriptoency regulating networks [16]. At the same time single-cell probes of gene expression in mESCs reveal significant heterogeneity and dynamism in bio-molecular populations [7, 8], suggesting that molecular stochasticity and non-equilibrium processes could be playing a crucial role in regulating pluripotnecy and stem cell differentiation. Thus the schematic network topology models inferred from real data, while immensely useful, are not always sufficient for rationalizing the single cell data, which is rich with stochastic and dynamical information. On the other hand, full chemical master equation based stochastic simulations of complex ESC networks with complete bimolecular inventory quickly become impractical, especially when making data-driven parameter searches and explorative simulations with a large number of external conditions.

In the present work we have developed a multi-scale computational scheme for converting experimentally-inferred Boolean topologies into quantitative and predictive models of networks with a microscopic resolution of gene expression dynamics. The employed computational model is based on previously proposed hybrid stochastic dynamics approaches [18, 23, 24, 35] in which the switching dynamics of individual genes are considered exactly while the rest of the biochemical reactions are approximated as deterministic processes. This hybrid-stochastic approach is approximately thousand fold faster than conventional kinetic Monte Carlo methods. This gain in computational speed allows us to simulate large scale gene regulatory networks of ESC under different culture conditions and gene-switching regimes. Thanks to rapid hybrid simulations we are able to use a standard optimization approach to exhaustively sample the space of rates and find the closest match with the experimental gene expression data collected different culturing environments [16]. The approximation introduced due to using the hybrid scheme is validated by showing excellent quantitative agreement of both steady states distributions and dynamical transition times with the fully stochastic simulations for the identified parameter sets. This agreement also shows that the switching events of genes—due to stochastic TFs binding to the promoter sites—is likely a dominant source of variance in transcription factor populations of ESC networks. Furthermore we argue that changes in gene-switching rates are a proxy for global epigenetic modifications which can alter the rates of access of transcription factors to sites buried under chromatin structures [76]. Thus, the significant remodeling of steady-state landscapes that we see by varying the global gene-switching rates suggests a powerful role for the global epigenetic changes in maintaining the stability of pluripotent states.

We find that the intermediate regime, in which the gene-switching rate is comparable to the other reaction rates in the network, is most consistent with single-cell measurements [7–9]. This result has also been pointed out by Sasai *et al*. [43] when exploring time-scale hierarchy in stem cell network with individual-based models and concluding that experimentally observed phenotypic heterogeneity likely originates from promoter reorganization and genetic switching taking place on the comparable time-scale with the rest of the biochemical processes. In this intermediate switching regime the transcription factors show bursty dynamics which lead to heterogeneous distributions with some showing long-tailed and bimodal features. Consistent with many experiments [7–9], our simulations show that the presence of LIF and 2i signals is crucial for maintaining the stability of pluripotent states, which in our model are defined as states with an up-regulated triad of pluripotency factors Nanog/Oct4/Sox2. Withdrawal of either LIF/2i initiates lineage commitment via a robust pattern of reduced expression of the Nanog/Oct4/Sox2 triad in the simulations.

To characterize the dynamics of lineage commitment, we have computed the transition times of going from pluripotent to differentiated steady states. We find that slower gene switching generates more heterogeneous distributions of transcription factors, which in turn makes the network more responsive to changes in signaling conditions. For instance, the response time to LIF/2i withdrawal is much faster in a more stochastic regime than in the more deterministic regime corresponding to faster gene switching time scales. Next, by carrying out principal component analysis on ensembles of gene expression profiles, we find a much simpler description of pluripotency and lineage commitment in terms of effective probability landscapes. As the signals safeguarding pluripotency are removed, these landscapes reveal a gradual narrowing of the steady-state attractor explored by the network. Thus, we see a hierarchical organization of differentiation landscapes where pluripotent states pose the largest attractor which is maintained through the extracellular signals and the molecular noise of gene switching.

Given the rapid rise of information from high throughput single-cell nucleic acid based techniques (RNA-seq, RNA-FISH, qPCR, etc.), we expect the microscopic resolution models, reported in the present work, to play important roles in bridging the systems-level behavior of genetic networks with the underlying molecular-level processes of binding, reaction and diffusion.

## Methods

### Constructing the piecewise-deterministic Markov process (PDMP)

In the individual-based description of complex genetic networks studied in the present work, one models each individual reactive events as a Markov jump processes. The underlying master equation governing the Markvoian evolution of the entire network is analytically intractable and in general even numerical simulations quickly become computational inefficient once dimensionality of the system becomes too high [77]. Specifically what contributed to this inefficiency is the population scale of transcription factors for which it is common to have values on the order of Ω = 10^4^ as is characteristic for biological cells. Thus the use of standard continuous-time Monte Carlo [17, 78] sampling techniques becomes unfeasible especially if one wants to sample the kinetic parameter regimes for finding optimal set of rate coefficients.

Fortunately the latest efforts of modeling gene expression dynamics [18, 23, 24, 35, 63, 64, 79] have lead to the emergence of a new class of techniques which are broadly based on using a piecewise-deterministic Markov process (PDMP) to approximate the individual-based model with a switching property. In this section, we briefly recapitulate the construction of the PDMP. A more thorough analysis can be found in the literature cited [18, 23, 24, 35, 63, 64, 79].

A PDMP is a process such that, in between discrete random switching events, the evolution of the process is deterministic. To construct the deterministic evolution of the TF populations, starting from the chemical master equations, we performed Kramers–Moyal expansion [77, 80] in the population of TFs while maintaining the discreteness of the genetic state; we keep only the first order of the expansion. The result is a standard Liouville equation governing the *deterministic flow* of the distribution. The joint probability distribution of our model converges to the deterministic flow in a given genetic state and in the thermodynamic limit Ω → ∞ [80]. With the PDMP approach, the demographic noise originating from random birth-death events are neglected, so that the population density *x*_*i*_(*t*) of each TF evolves according to

$$\begin{array}{c} \frac{d}{dt}x_{i}\left(t\right)=\alpha_{i}-\gamma x_{i}\left(t\right), \end{array}$$ (3)

where *α*_*i*_ ∈ {0, *α*_*m*_, *α*_max_} is the production rate of the *i*th TF dependent on the *i*th gene’s configuration of promoter sites. While the evolution of the TF population density is deterministic, the binding and unbinding events of the regulating TFs to their target genes are still stochastic and formulated according to Eq 1 in the main text.

We finally emphasize that the PDMP only retains the contribution of *switching noise* which arise from the discrete and stochastic binding and unbinding events between the TFs and the promoter sites, and ignores *demographic stochasticity* from the discrete production and degradation processes of the TFs. The PDMP is the limiting process when the population scale Ω → ∞ [18], and the error bound of the description can be rigorously derived to be $O(\Omega^{-1})$ [81].

### Generating exact sample paths of the PDMP

To simulate the stochastic binding and unbinding statistics of the promoter sites, accurate waiting times must be generated. A waiting time exists for each possible stochastic transition; the smallest of these times tells us how long the system stays in the current configuration of promoter sites, and to which promoter configuration it transitions. In general, waiting times can be generated by mapping a uniform random variable to a random time using the survival function. Since in our case the transition rates are functions of dynamical state variables, this involves the numerical integration of survival functions describing each potential transition [63].

In our case, the simple form of Eq 3 (and thus of the transition rates) allows us to improve upon this by generating waiting times without numerical integration, detailed in the Supplementary Information.

### Non-dimensionalization of model parameters

Under the assumptions we proposed, there are initially six free model parameters: Ω*α*_max_ and Ω*α*_*m*_ as the production rates when each of the genes has an “ON” or “MEDIUM” activity, *γ* as the protein degradation rate, *N* as the number of promoter sites, and lastly *k*_on_Ω^−1^ and *k*_off_ as the binding and unbinding rates between the TFs and the promoter sties. We remark that the population scale Ω is fixed at 10^4^.

Through suitable non-dimensionalization of the physical time and concentrations of the TFs, we reduce the number of parameters. As can be seen from the above formulation (Eq 3), the time scale of the TF dynamics is set by the degradation rate *γ*. For stable proteins, the time scale of degradation is of the order of the times of the cell cycle. We therefore choose the unit of physical time such that *γ* is 1. Similarly, the maximum concentration in the TF can achieve in Eq 3 is *α*_max_/*γ*. We can choose a unit for the concentrations of the chemical species such that *α*_max_ = 1, so the concentration of the TFs are always bounded in (0, 1). After non-dimensionalization, the model ends up with four free parameters: *α*_*m*_ ∈ {0, 1} as the intermediate production rate of those genes which are regulated by both activators and repressors, *k*_on_, *k*_off_ as the binding and unbinding rate of the TF to the promoter sites, and *N* as the number of promoter sites per gene. We use these non-dimensionalized parameters to report our results in the manuscript.

### Using the checkerboard diagram to infer the parameter regime

To narrow down the parameter regime, we match our model predictions to the experimental findings of Dunn *et al.* [16] in which the authors measured the TF expression under various combinations of external signals, i.e., LIF, CH, and PD. We aim to match the model prediction to a twelve-by-five “checkerboard diagram” which records the experimentally measured expression pattern presented in Fig 2 in the main text. To achieve this goal, we performed a sweep in a vast parameter space: *α*_*m*_ ∈ [0, 1], *k*_on_, *k*_off_ ∈ [0, 110], and *N* = 1, 2 … 5. For each parameter set, we simulated 10^3^ PDMP sample paths for a time to sufficiently reflect the stationary state, and the average TF expression levels were recorded. Because of the non-dimensionalization, the expression level (the population density) of each TF is a real number in between 0 and 1. This results in a twelve-by-five real-valued matrix, which is binarized by a threshold. To find the optimal threshold, we use the number of discrepancies between the model prediction and the target matrix—the Hamming distance—as a quantitative measure. For each parameter set, an optimal threshold which minimizes the Hamming distance was then found computationally, and the minimal Hamming distance was recorded and plotted in Fig 2 in the main text as a “landscape” of how good the model captures the experimental results. We found that for *N* = 1 and *N* ≥ 2, the global minimal Hamming distance is 5 and 3 respectively. We chose *N* = 2 to present our follow-up analysis, as it incorporates the capacity of modeling cooperative binding which is often modeled phenomenologically. We find the Hamming distance can be constantly as small as 3 in a vast region in the space of binding/unbinding rates when *α*_*m*_ is small (≲ 0.02). Therefore, in the manuscript we present the landscape of a fast switching regime *k*_on_ ≈ 100, an intermediate regime *k*_on_ ≈ 15 and a slow switching regime *k*_on_ ≈ 3.

### Validating the PDMP against the individual-based model

For the three selected parameter sets, 10^4^ sample paths of a fully individual-based model were generated by standard kinetic Monte Carlo simulations—namely Gillespie’s stochastic simulation algorithm (SSA) [17, 78]. The population scale Ω for each TF is set to be 10^4^. A parallel analysis is carried out and the results are consistent with the predictions from using the PDMP. We report the results of the intermediate switching regime in Fig 4 of the main text.

### Visualizing stochatic fluctuations in gene expression on low dimensional manifolds using principal component analysis (PCA)

While the joint probability distributions are measured by kinetic Monte Carlo sampling, the dimensionality of the dynamical system is very high: each TF has a real-valued density, so that even if we marginalize over the genetic states the probability density is a 12-dimensional object. Although Fig 4 in the main text summarizes the marginal distributions of the real-valued TF density and contains rich information, it is desirable to visualize the results in a lower dimensional space to draw qualitative conclusions. To achieve this goal, we perform the standard principal component analysis [82]. We chose a baseline external condition to be LIF+2i; the first two principal components were computed. For the rest of the external conditions, the joint probability distributions are projected onto the plane spanned by these principal components; the results are presented in Fig 7 in the main text.

### Dynamical transitions between different external signals

To investigate dynamical transitions when the external driving conditions (whether LIF, CH, and PD are present) change, we prepare 10^5^ independent sample paths with an initial external condition until the joint probability distribution converges to the stationary distribution. Then, the external condition is switched instantaneously to the second condition. We further evolve the dynamical system until stationarity for the second conditions is reached. The results are summarized in Fig 5 of the main text. To estimate the transition times between the stationary distributions with different external conditions, we measure the Jensen–Shannon distance of the marginal distribution of each TF density, at any given time during the transition to the final marginal distribution. We measure and report the first time when all 12 distances are below a threshold value of 0.3, presented in Fig 6 of the main text.

### Numerical implementation

Both the PDMP and the IB models have been implemented in c++, using the algorithm presented in the Supporting Information and the standard kinetic Monte Carlo algorithm [17, 78] respectively. The network topology was hard-coded in the simulators to improve the simulation efficiency. The simulators are provided in the Supporting information. To facilitate the use of our computational work and ensure full reproducibility, the IB model is translated into standardized BioNetGen language [83, 84] and provided in Supporting Information, along with the standard SBML format of the model exported by BNGL.

## Supporting information

S1 Text Supplementary information. (PDF)

S1 File c++ source code of the kinetic Monte Carlo simulator to generate exact distributions of the PDMP; SBML and BNGL files for numerically implementing individual based model of the pluripotency network. (ZIP)

S1 Table The transition times (normalized by 1/*γ* ≈ 8*hr*) in the fast switching regime. (PDF)

S2 Table The transition times (normalized by 1/*γ* ≈ 8*hr*) in the intermediate switching regime. (PDF)

S1 Fig Pairwise correlations between different transcription factors under signaling conditions LIF+2i, LIF, 2i and None. We show the results for the fast, intermediate, and slow-switching parameter regimes defined in the manuscript.
(PDF)

S2 Fig (N = 1) The Hamming distance (shown in color) as function of model parameters *α*_*m*_, *k*_*on*_ (x-axis of the figures), and *k*_*off*_ (y-axis of the figures). Each column corresponds to one of the three (slow-, intermediate- and fast-switching) regimes.
(PNG)

S3 Fig (N = 2) The Hamming distance (shown in color) as function of model parameters *α*_*m*_, *k*_*on*_ (x-axis of the figures), and *k*_*off*_ (y-axis of the figures). Each column corresponds to one of the three (slow-, intermediate- and fast-switching) regimes.
(PNG)

S4 Fig (N = 3) The Hamming distance (shown in color) as function of model parameters *α*_*m*_, *k*_*on*_ (x-axis of the figures), and *k*_*off*_ (y-axis of the figures). Each column corresponds to one of the three (slow-, intermediate- and fast-switching) regimes.
(PNG)

S5 Fig (N = 4) The Hamming distance (shown in color) as function of model parameters *α*_*m*_, *k*_*on*_ (x-axis of the figures), and *k*_*off*_ (y-axis of the figures). Each column corresponds to one of the three (slow-, intermediate- and fast-switching) regimes.
(PNG)

S6 Fig (N = 5) The Hamming distance (shown in color) as function of model parameters *α*_*m*_, *k*_*on*_ (x-axis of the figures), and *k*_*off*_ (y-axis of the figures). Each column corresponds to one of the three (slow-, intermediate- and fast-switching) regimes.
(PNG)

S7 Fig The TF expression pattern (in both continuous space and binarized form) for different number of promoter sites N = 1, 2, 3, 4, and 5. The expression patterns were generated with the parameter set (*α*_*m*_, *k*_*on*_, *k*_*off*_) which has the minimal Hamming distance among the points we sampled in Figs. S2-S7 to the experimental data in Dunn et al. Each column corresponds to one of the three (slow-, intermediate- and fast-switching) regimes.
(PNG)

S8 Fig PDMP faithfully captures the dynamics of the individual-based model. First row: reproduction of Fig 4 of the main text manuscript. Second row: corresponding IB model (with ∼ 100 sample paths) with the same perturbations.
(PNG)
