# Supplementary material for: A stochastic and dynamical view of pluripotency in mouse embryonic stem cells
Source: PLoS Comput Biol. 2018 Feb 16;14(2):e1006000. doi: 10.1371/journal.pcbi.1006000 (PMC5833290; doi:10.1371/journal.pcbi.1006000)
Supplement: S1 Text — (PDF) [file pcbi.1006000.s001.pdf]

Appendix S1 to "A stochastic and dynamical view of pluripotency in mouse embryonic stem cells"

Yen Ting Lin,[1, 2] Peter G. Hufton,[2] Esther J. Lee,[3] and Davit A. Potoyan[4]

[1] *Theoretical Division and Center for Nonlinear Studies, Los Alamos National Laboratory, Los Alamos, NM 87545, USA*
[2] *School of Physics and Astronomy, The University of Manchester, Manchester, M13 9PL, UK*
[3] *Department of Bioengineering, Rice University, Houston, TX 77005, USA*
[4] *Department of Chemistry, Iowa State University, Ames, IA 50011, USA*

(Dated: February 1, 2018)

I. CONSTRUCTING THE PIECEWISE-DETERMINISTIC MARKOV PROCESS (PDMP)

In the individual-based description of complex genetic networks studied in the present work, one models each individual reactive events as a Markov jump processes. The underlying master equation governing the Markvoian evolution of the entire network is analytically intractable and in general even numerical simulations quickly become computational inefficient once dimensionality of the system becomes too high[1]. Specifically what contributed to this inefficiency is the population scale of transcription factors for which it is common to have values on the order of $\Omega = 10^4$ as is characteristic for biological cells. Thus the use of standard continuous-time Monte Carlo [2, 3] sampling techniques becomes unfeasible especially if one wants to sample the kinetic parameter regimes for finding optimal set of rate coefficients.

Fortunately the latest efforts of modeling gene expression dynamics [4–10] have lead to the emergence of a new class of techniques which are broadly based on using a piecewise-deterministic Markov process (PDMP) to approximate the individual-based model with a switching property. In this section, we briefly recapitulate the construction of the PDMP. A more thorough analysis can be found in the literature cited [4–10] .

A PDMP is a process such that, in between discrete random switching events, the evolution of the process is deterministic. To construct the deterministic evolution of the TF populations, starting from the chemical master equations, we performed Kramers–Moyal expansion [1, 11] in the population of TFs while maintaining the discreteness of the genetic state; we keep only the first order of the expansion. The result is a standard Liouville equation governing the *deterministic flow* of the distribution. The joint probability distribution of our model converges to the deterministic flow in a given genetic state and in the thermodynamic limit $\Omega \to \infty$ [11]. With the PDMP approach, the demographic noise originating from random birth-death events are neglected, so that the population density $x_i(t)$ of each TF evolves according to

$$\frac{d}{dt}x_i(t) = \alpha_i - \gamma x_i(t), \tag{1}$$

where $\alpha_i \in \{0, \alpha_m, \alpha_{\max}\}$ is the production rate of the ith TF dependent on the ith gene's configuration of promoter sites. While the evolution of the TF population density is deterministic, the binding and unbinding events of the regulating TFs to their target genes are still stochastic and formulated according to Eq. 1 in the main text.

We finally emphasize that the PDMP only retains the contribution of *switching noise* which arise from the discrete and stochastic binding and unbinding events between the TFs and the promoter sites, and ignores *demographic stochasticity* from the discrete production and degradation processes of the TFs. The PDMP is the limiting process when the population scale $\Omega \to \infty$ [7], and the error bound of the description can be rigorously derived to be $\mathcal{O}(\Omega^{-1})$ [12].

II. GENERATING EXACT SAMPLE PATHS OF THE PDMP

To simulate the stochastic binding and unbinding statistics of the promoter sites, accurate waiting times must be generated. A waiting time exists for each possible stochastic transition; the smallest of these times tells us how long the system stays in the current configuration of promoter sites, and to which promoter configuration it transitions. In general, waiting times can be generated by mapping a uniform random variable to a random time using the survival function. Since in our case the transition rates are functions of dynamical state variables, this involves the numerical integration of survival functions describing each potential transition [8].

In our case, the simple form of Eq. 1 (and thus of the transition rates) allows us to improve upon this by generating waiting times without numerical integration, detailed in the next section.

III. EFFICIENT GENERATION OF WAITING TIMES FOR THE GENETIC SWITCHING

Our approach requires the generation of accurate waiting times, which dictate how long the system stays in the current configuration of promoter sites, and to which promoter configuration it transitions. In our case the simple form of the PDMP, and consequently of the transition rates, allows us to generating waiting times without numerical integration of the survival function.

The TF density of a given type, with an initial condition x_0, is described by

$$x(t) = \frac{\alpha}{\gamma} + \left(x\left(t=0\right) - \frac{\alpha}{\gamma}\right) \exp\left(-\gamma t\right). \tag{2}$$

We dropped the subscript i for brevity in this section, as all the TFs will be evolving according to the same equation (but with different α which is determined by the promoter states.) It follows that the survival function—the probability that the switching time is greater than time t—describing a genetic binding event is given by

$$\begin{aligned} S(t) &= \exp\left[-k_{\text{on}} \int_0^t x(t')\,\mathrm{d}t'\right] \\ &= \exp\left\{-\frac{k_{\text{on}}}{\gamma}\left[\alpha t - \left(x\left(t=0\right) - \frac{\alpha}{\gamma}\right)\left(e^{-\gamma t} - 1\right)\right]\right\}. \end{aligned} \tag{3}$$

To use the inverse method, one generates a random number $u \sim \text{Unif}\left(0,1\right)$ and solves the equation $u = S(t)$ for t. The solution is a random binding time with the correct distribution.

When the density is monotonically decreasing ($x_0 > \alpha$), the procedure allows one to rigorously generate *exact* switching times [13]. This involves generating two independent random numbers u_1, $u_2 \sim \text{Unif}\left(0,1\right)$ such that the random time of a binding event t is given by $t = \min(t_1, t_2)$ where

$$t_1 = \begin{cases} -\gamma\left(\log u_1\right)/\left(\alpha k_{\text{on}}\right) & \text{if } \alpha \neq 0, \\ \infty & \text{if otherwise.} \end{cases} \tag{4a}$$

$$t_2 = \begin{cases} -\gamma \log\left\{\left(\log u_2\right)/\left[k_{\text{on}}\left(x_0 - \alpha/\gamma\right)\right] + 1\right\} & \text{if } u_2 > \exp\left[-k_{\text{on}}\left(x_0 - \alpha/\gamma\right)\right], \\ \infty & \text{if otherwise.} \end{cases} \tag{4b}$$

The case when the density is monotonically increasing $u = S(t)$ is not analytically solvable but can be solved numerically and with efficiency using the Newton–Raphson scheme. Using these two approaches together, the random waiting times for the next binding event on gene i can be efficiently sampled.

The unbinding events are independent of the population of TFs. Since each bound TF on a promoter dissociate independently and identically with a rate k_{off}, the waiting time of each of the dissociating events is exponentially distributed ($\sim \text{Exp}\left(k_{\text{off}}\right)$) and can be efficiently generated [2].

At any given point of time and given the state of the system, we can use the above procedures to generate the random waiting times for binding or unbinding events. Before the first event (i.e., the binding or unbinding event with the minimal waiting times) takes place, the dynamics of TF evolve deterministically and all the promoter states remains still. At the time of the next binding or unbinding event, the promoter state corresponding to the fist binding or unbinding event is updated, and the random waiting times needs to be updated.

IV. NON-DIMENSIONALIZATION OF MODEL PARAMETERS

Under the assumptions we proposed, there are initially six free model parameters: $\Omega\alpha_{\text{max}}$ and $\Omega\alpha_m$ as the production rates when each of the genes has an "ON" or "MEDIUM" activity, γ as the protein degradation rate, N as the number of promoter sites, and lastly $k_{\text{on}}\Omega^{-1}$ and k_{off} as the binding and unbinding rates between the TFs and the promoter sties. We remark that the population scale Ω is fixed at 10^4.

Through suitable non-dimensionalization of the physical time and concentrations of the TFs, we reduce the number of parameters. As can be seen from the above formulation (Eq. 1), the time scale of the TF dynamics is set by the degradation rate γ. For stable proteins, the time scale of degradation is of the order of the times of the cell cycle. We therefore choose the unit of physical time such that γ is 1. Similarly, the maximum concentration in the TF can achieve in Eq. 1 is $\alpha_{\text{max}}/\gamma$. We can choose a unit for the concentrations of the chemical species such that $\alpha_{\text{max}} = 1$, so the concentration of the TFs are always bounded in $(0, 1)$. After non-dimensionalization, the model ends up with four free parameters: $\alpha_m \in \{0, 1\}$ as the intermediate production rate of those genes which are regulated by both activators and repressors, k_{on}, k_{off} as the binding and unbinding rate of the TF to the promoter sites, and N as the number of promoter sites per gene. We use these non-dimensionalized parameters to report our results in the manuscript.

V. USING THE CHECKERBOARD DIAGRAM TO INFER THE PARAMETER REGIME

To narrow down the parameter regime, we match our model predictions to the experimental findings of Dunn *et al.* [14] in which the authors measured the TF expression under various combinations of external signals, i.e., LIF, CH, and PD. We aim to match the model prediction to a twelve-by-five "checkerboard diagram" which records the experimentally measured expression pattern presented in Fig. 2 in the main text. To achieve this goal, we performed a sweep in a vast parameter space: $\alpha_m \in [0,1]$, $k_{\text{on}}, k_{\text{off}} \in [0,110]$, and $N = 1, 2 \ldots 5$. For each parameter set, we simulated 10^3 PDMP sample paths for a time to sufficiently reflect the stationary state, and the average TF expression levels were recorded. Because of the non-dimensionalization, the expression level (the population density) of each TF is a real number in between 0 and 1. This results in a twelve-by-five real-valued matrix, which is binarized by a threshold. To find the optimal threshold, we use the number of discrepancies between the model prediction and the target matrix—the Hamming distance—as a quantitative measure. For each parameter set, an optimal threshold which minimizes the Hamming distance was then found computationally, and the minimal Hamming distance was recorded and plotted in Fig. 2 in the main text as a "landscape" of how good the model captures the experimental results.We found that for $N = 1$ and $N \geq 2$, the global minimal Hamming distance is 5 and 3 respectively. We chose $N = 2$ to present our follow-up analysis, as it incorporates the capacity of modeling cooperative binding which is often modeled phenomenologically. We find the Hamming distance can be constantly as small as 3 in a vast region in the space of binding/unbinding rates when α_m is small ($\lesssim 0.02$). Therefore, in the manuscript we present the landscape of a fast switching regime $k_{\text{on}} \approx 100$, an intermediate regime $k_{\text{on}} \approx 15$ and a slow switching regime $k_{\text{on}} \approx 3$.

VI. VALIDATING THE PDMP AGAINST THE INDIVIDUAL-BASED MODEL

For the three selected parameter sets, 10^4 sample paths of a fully individual-based model were generated by standard kinetic Monte Carlo simulations—namely Gillespie's stochastic simulation algorithm (SSA) [2, 3]. The population scale Ω for each TF is set to be 10^4. A parallel analysis is carried out and the results are consistent with the predictions from using the PDMP. We report the results of the intermediate switching regime in Fig. 4 of the main text.

VII. VISUALIZING STOCHATIC FLUCTUATIONS IN GENE EXPRESSION ON LOW DIMENSIONAL MANIFOLDS USING PRINCIPAL COMPONENT ANALYSIS (PCA)

While the joint probability distributions are measured by kinetic Monte Carlo sampling, the dimensionality of the dynamical system is very high: each TF has a real-valued density, so that even if we marginalize over the genetic states the probability density is a 12-dimensional object. Although Fig. 4 in the main text summarizes the marginal distributions of the real-valued TF density and contains rich information, it is desirable to visualize the results in a lower dimensional space to draw qualitative conclusions. To achieve this goal, we perform the standard principal component analysis [15]. We chose a baseline external condition to be LIF+2i; the first two principal components were computed. For the rest of the external conditions, the joint probability distributions are projected onto the plane spanned by these principal components; the results are presented in Fig. 7 in the main text.

VIII. DYNAMICAL TRANSITIONS BETWEEN DIFFERENT EXTERNAL SIGNALS

To investigate dynamical transitions when the external driving conditions (whether LIF, CH, and PD are present) change, we prepare 10^5 independent sample paths with an initial external condition until the joint probability distribution converges to the stationary distribution. Then, the external condition is switched instantaneously to the second condition. We further evolve the dynamical system until stationarity for the second conditions is reached. The results are summarized in Fig. 5 of the main text. To estimate the transition times between the stationary distributions with different external conditions, we measure the Jensen–Shannon distance of the marginal distribution of each TF density, at any given time during the transition to the final marginal distribution. We measure and report the first time when all 12 distances are below a threshold value of 0.3, presented in Fig. 6 of the main text.

[1] Gardiner CW, et al. Handbook of stochastic methods. vol. 3. Springer Berlin; 1985.

[2] Schwartz R. Biological modeling and simulation: a survey of practical models, algorithms, and numerical methods. MIT Press; 2008.

[3] Gillespie DT. Exact stochastic simulation of coupled chemical reactions. The journal of physical chemistry. 1977;81(25):2340–2361.

[4] Potoyan DA, Wolynes PG. Dichotomous noise models of gene switches. The Journal of chemical physics. 2015;143(19):195101.

[5] Lin YT, Galla T. Bursting noise in gene expression dynamics: linking microscopic and mesoscopic models. Journal of The Royal Society Interface. 2016;13(114):20150772.

[6] Lin YT, Doering CR. Gene expression dynamics with stochastic bursts: Construction and exact results for a coarse-grained model. Physical Review E. 2016;93(2):022409.

[7] Hufton PG, Lin YT, Galla T, McKane AJ. Intrinsic noise in systems with switching environments. Physical Review E. 2016;93(5):052119.

[8] Zeiser S, Franz U, Wittich O, Liebscher V. Simulation of genetic networks modelled by piecewise deterministic Markov processes. IET systems biology. 2008;2(3):113–135.

[9] Zeiser S, Franz U, Liebscher V. Autocatalytic genetic networks modeled by piecewise-deterministic Markov processes. Journal of Mathematical Biology. 2010;60(2):207–246.

[10] Lin YT, Buchler NE. Efficient analysis of stochastic gene dynamics in the non-adiabatic regime using piecewise deterministic Markov processes. ArXiv e-prints. 2017;.

[11] Kurtz TG. Solutions of ordinary differential equations as limits of pure jump Markov processes. Journal of applied Probability. 1970;7(1):49–58.

[12] Jahnke T, Kreim M. Error bound for piecewise deterministic processes modeling stochastic reaction systems. Multiscale Modeling & Simulation. 2012;10(4):1119–1147.

[13] Bokes P, King JR, Wood AT, Loose M. Transcriptional bursting diversifies the behaviour of a toggle switch: hybrid simulation of stochastic gene expression. Bulletin of mathematical biology. 2013;75(2):351–371.

[14] Dunn SJ, Martello G, Yordanov B, Emmott S, Smith A. Defining an essential transcription factor program for naive pluripotency. Science. 2014;344(6188):1156–1160.

[15] Jolliffe I. Principal component analysis. Wiley Online Library; 2002.
