# Supplementary material for: A stochastic and dynamical view of pluripotency in mouse embryonic stem cells
Source: PLoS Comput Biol. 2018 Feb 16;14(2):e1006000. doi: 10.1371/journal.pcbi.1006000 (PMC5833290; doi:10.1371/journal.pcbi.1006000)
Supplement: S1 Table — (PDF) [file pcbi.1006000.s003.pdf]

Initial state \ Final state	None	PD	CH	2i	LIF	LIF+PD	LIF+CH	LIF+2i
None	0.0	4.95	5.4	8.75	4.75	4.8	4.75	5.05
PD	4.85	0.0	4.5	5.85	4.9	4.7	4.9	4.85
CH	5.45	4.4	0.0	3.65	4.2	4.2	4.2	4.2
2i	12.4	6.7	4.5	0.0	4.5	4.2	4.5	4.2
LIF	18.5	11.5	9.5	8.55	0.0	3.5	1.25	2.95
LIF+PD	18.0	11.1	9.2	8.55	4.5	0.0	4.5	2.45
LIF+CH	18.65	11.55	9.65	8.55	0.75	3.5	0.0	2.95
LIF+2i	18.45	11.3	9.65	8.55	4.5	1.75	4.5	0.0

TABLE S1. The transition times (normalized by $1/\gamma \approx 8$ hr) in the fast switching regime.
