# Supplementary figures and images for: A stochastic and dynamical view of pluripotency in mouse embryonic stem cells
Source: PLoS Comput Biol. 2018 Feb 16;14(2):e1006000. doi: 10.1371/journal.pcbi.1006000 (PMC5833290; doi:10.1371/journal.pcbi.1006000)

Fast switching

LIF+2i

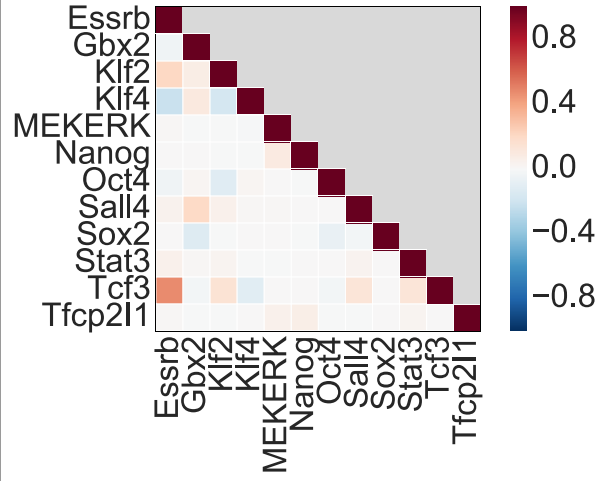

LIF

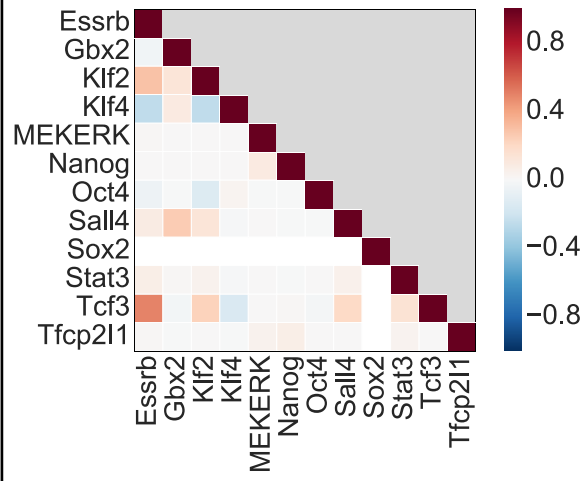

2i

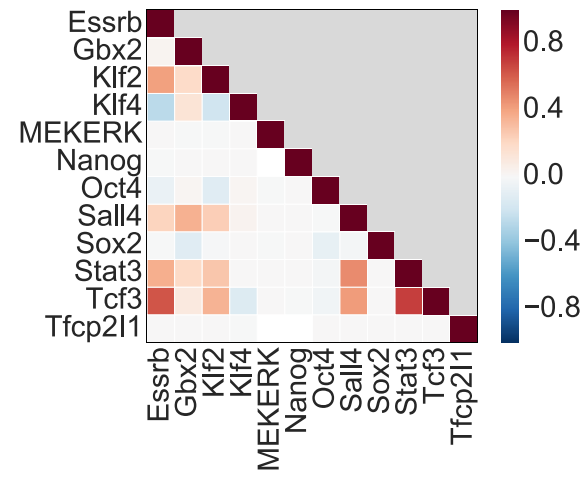

None

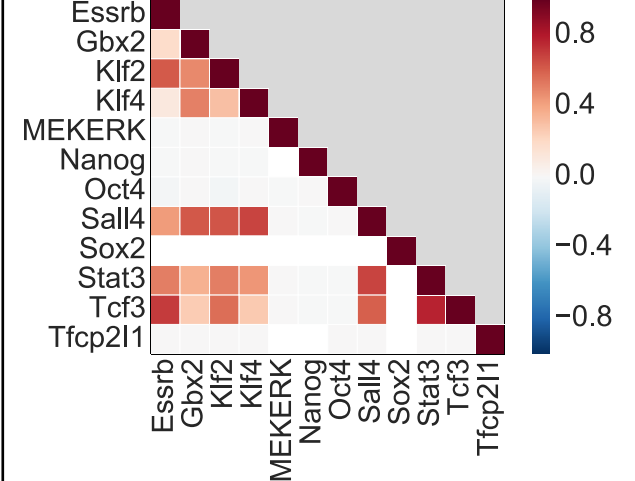

Intermediate switching

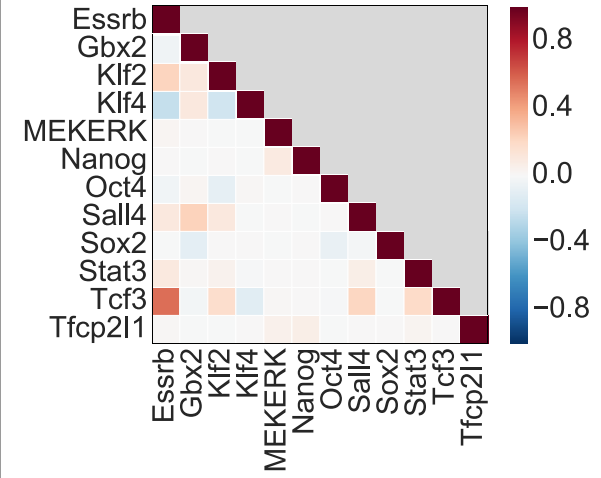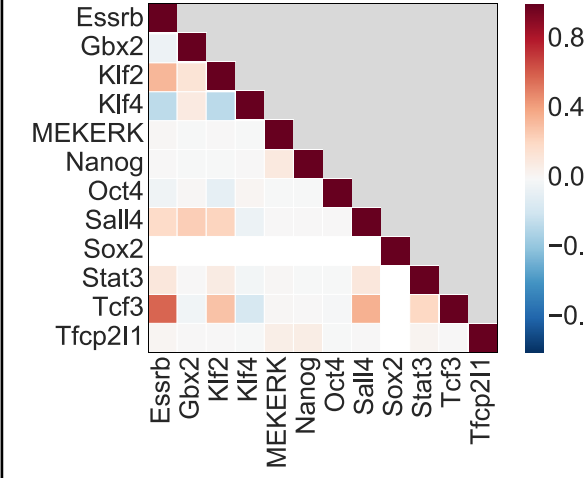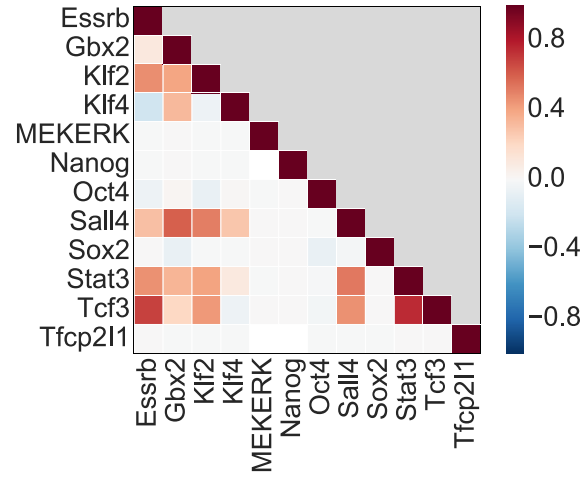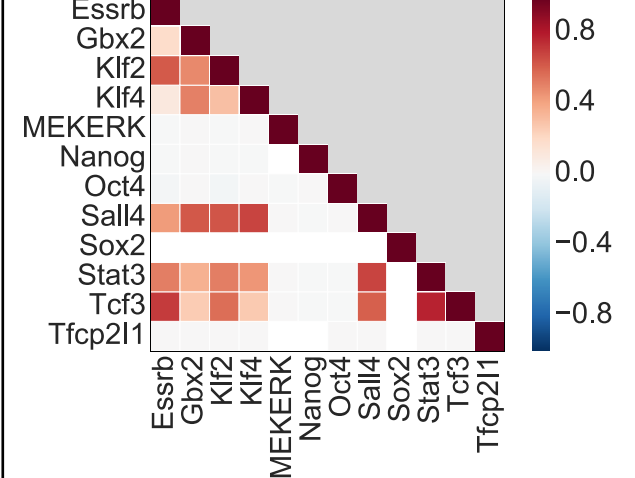

Slow switching

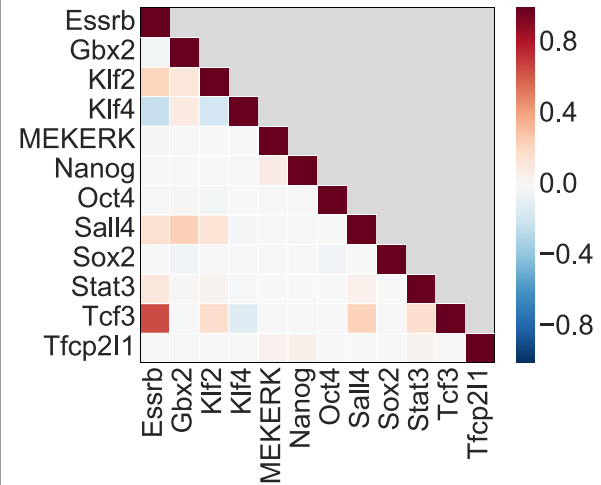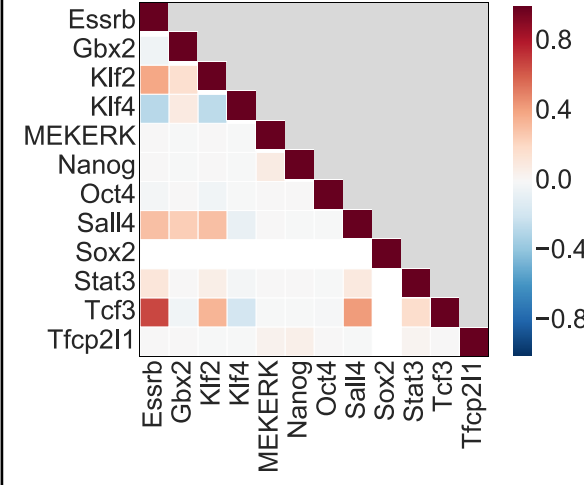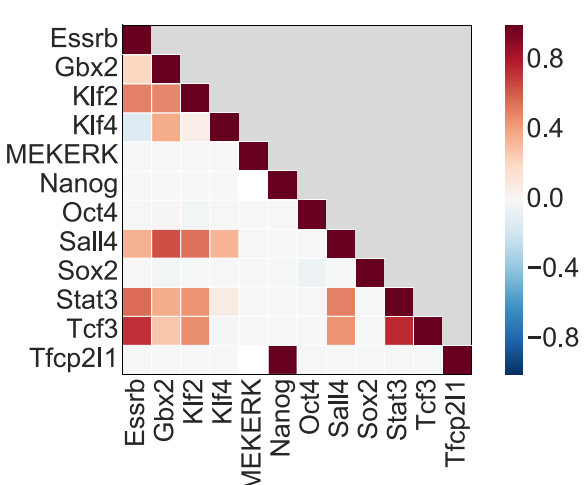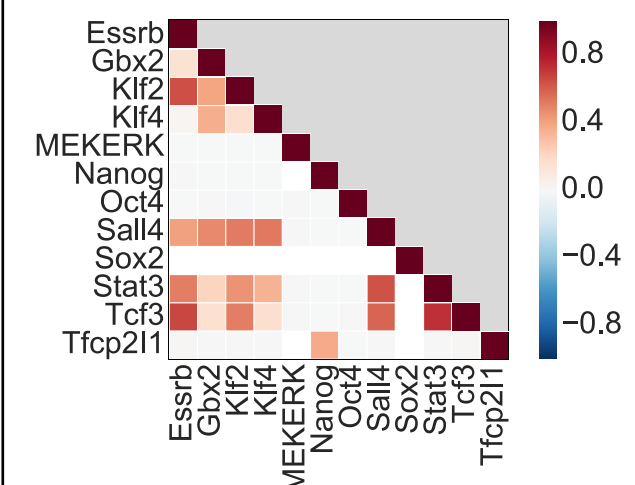

Supplement: S1 Fig — We show the results for the fast, intermediate, and slow-switching parameter regimes defined in the manuscript. (PDF) [file pcbi.1006000.s005.pdf]

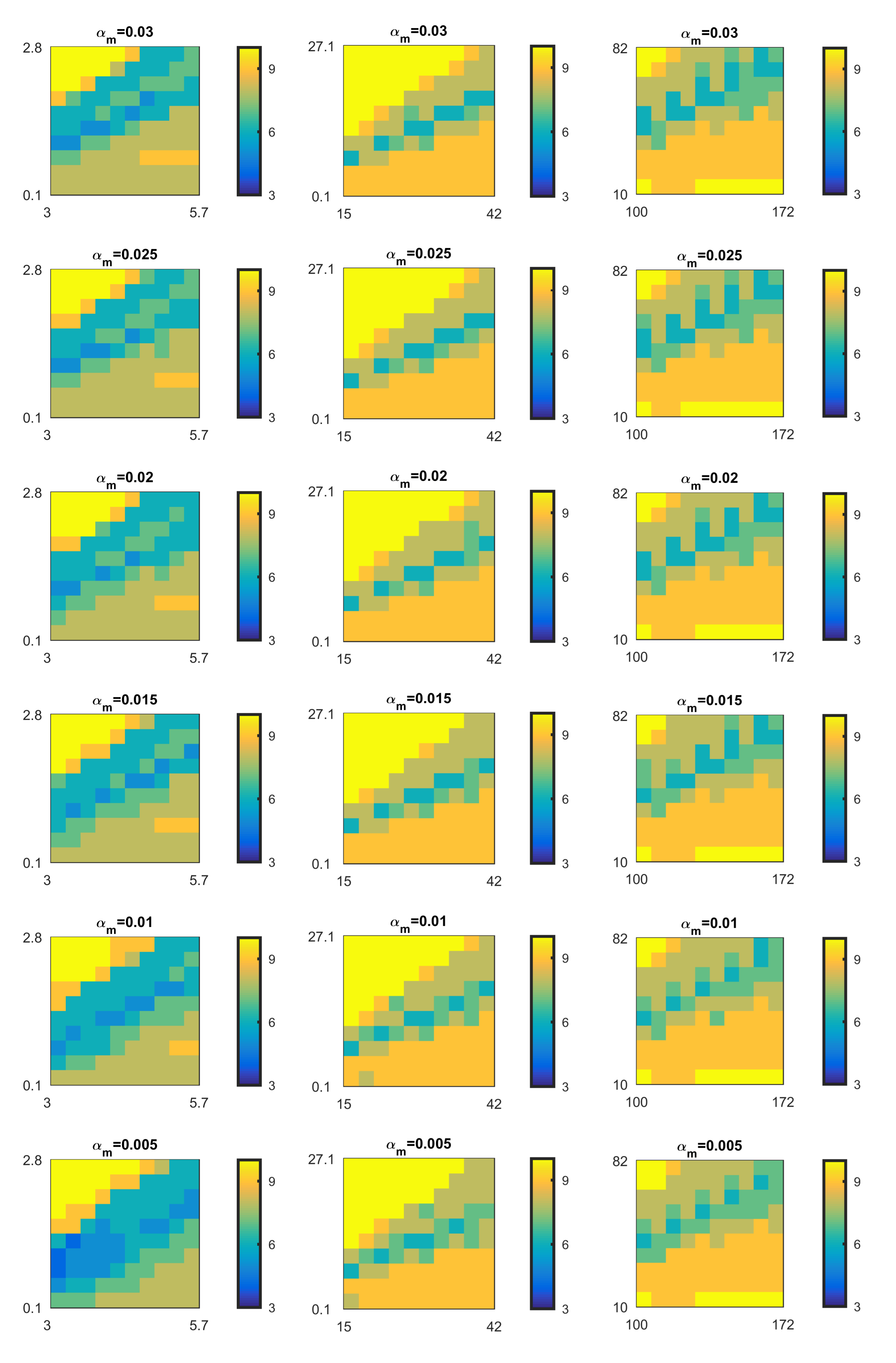

Supplement: S2 Fig — Each column corresponds to one of the three (slow-, intermediate- and fast-switching) regimes. (PNG) [file pcbi.1006000.s006.png]

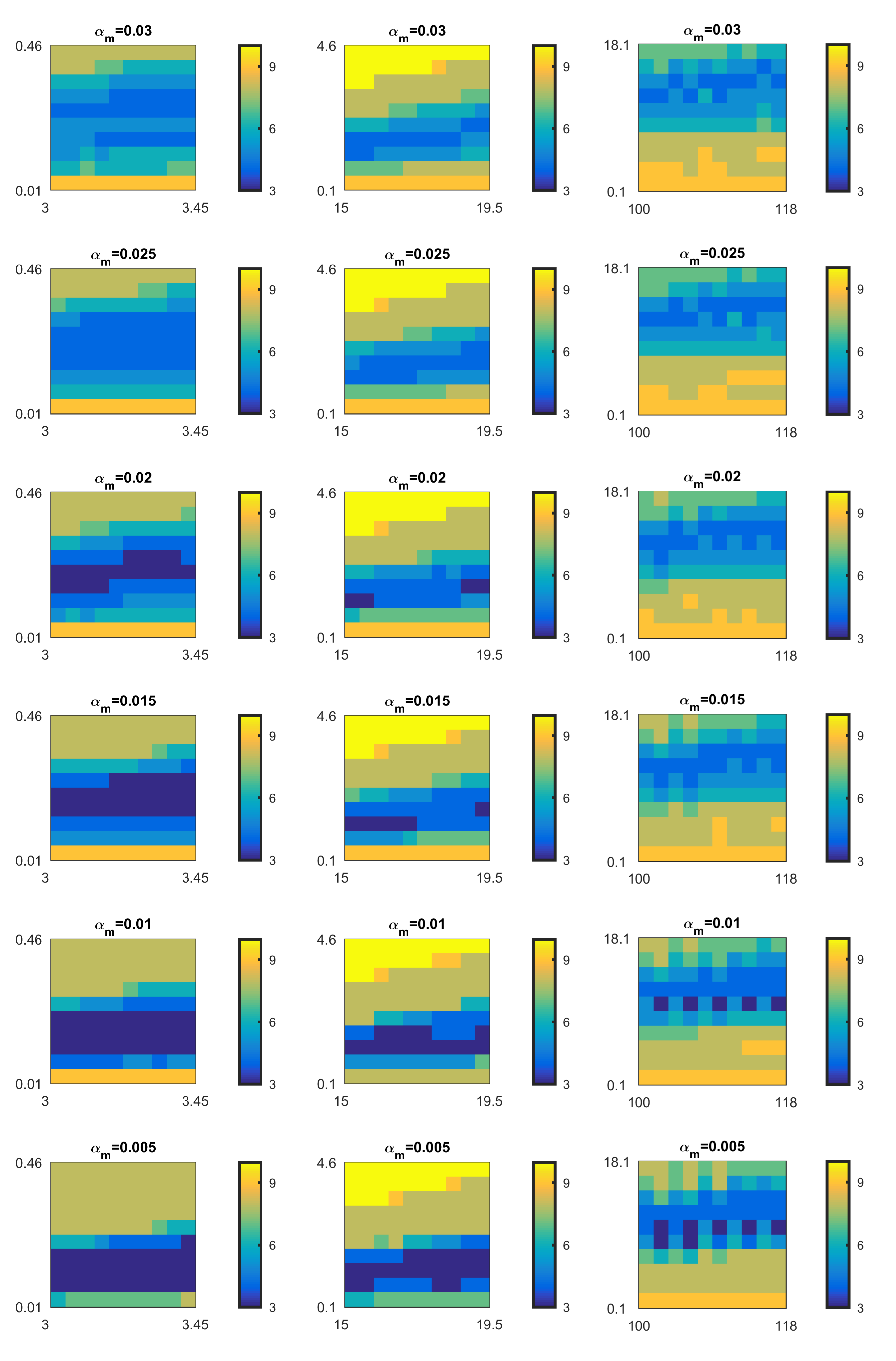

Supplement: S3 Fig — Each column corresponds to one of the three (slow-, intermediate- and fast-switching) regimes. (PNG) [file pcbi.1006000.s007.png]

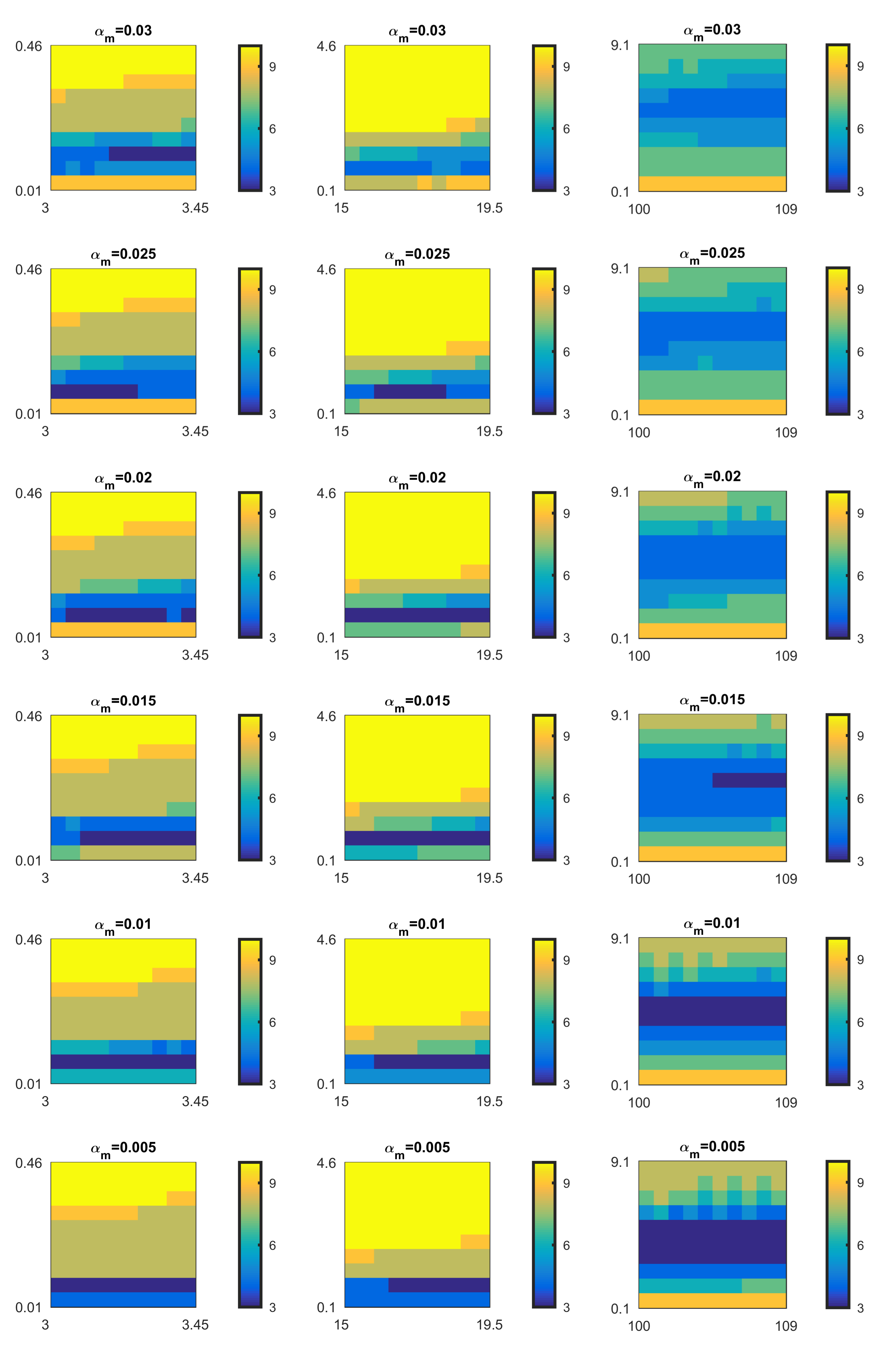

Supplement: S4 Fig — Each column corresponds to one of the three (slow-, intermediate- and fast-switching) regimes. (PNG) [file pcbi.1006000.s008.png]

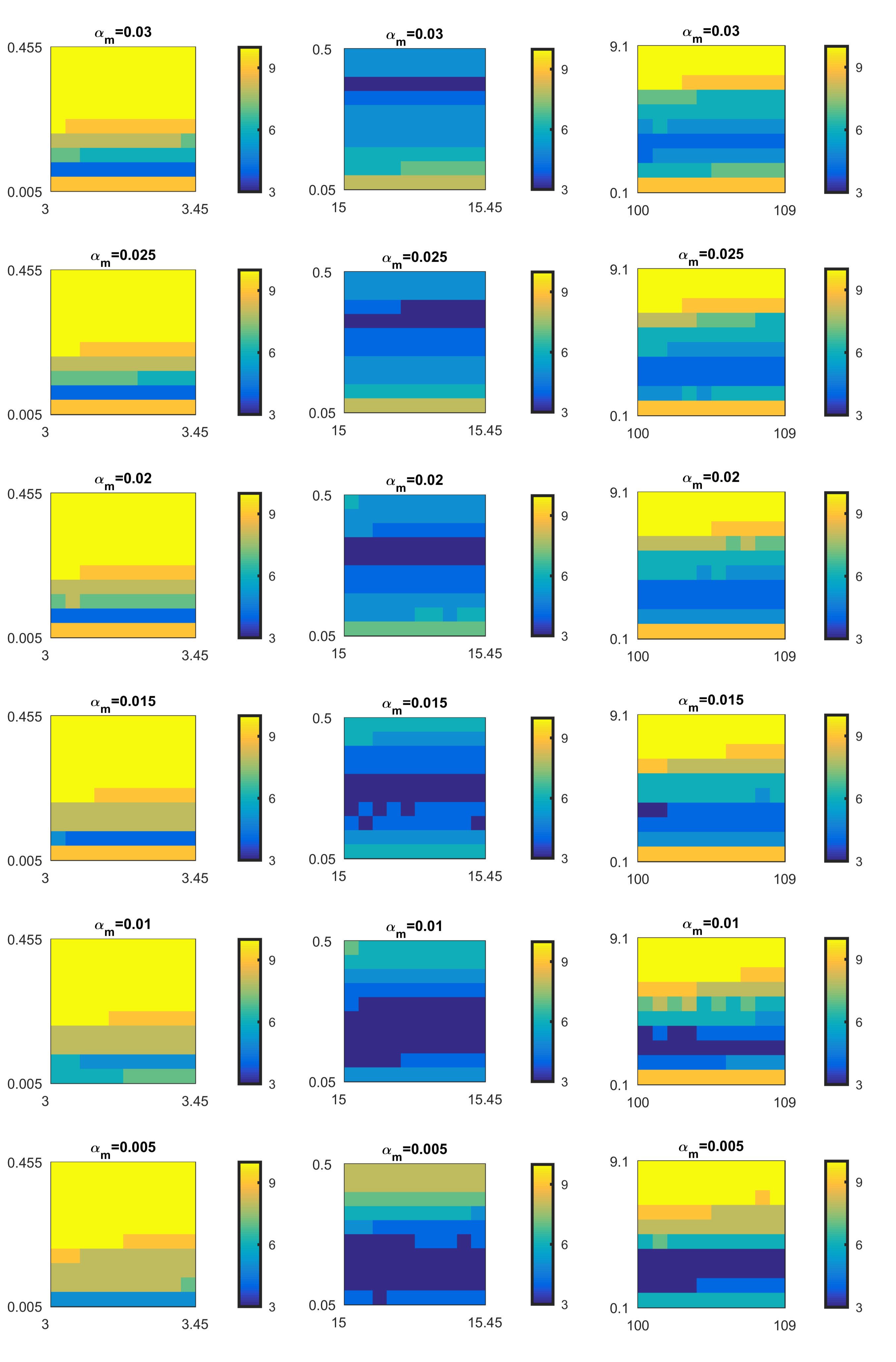

Supplement: S5 Fig — Each column corresponds to one of the three (slow-, intermediate- and fast-switching) regimes. (PNG) [file pcbi.1006000.s009.png]

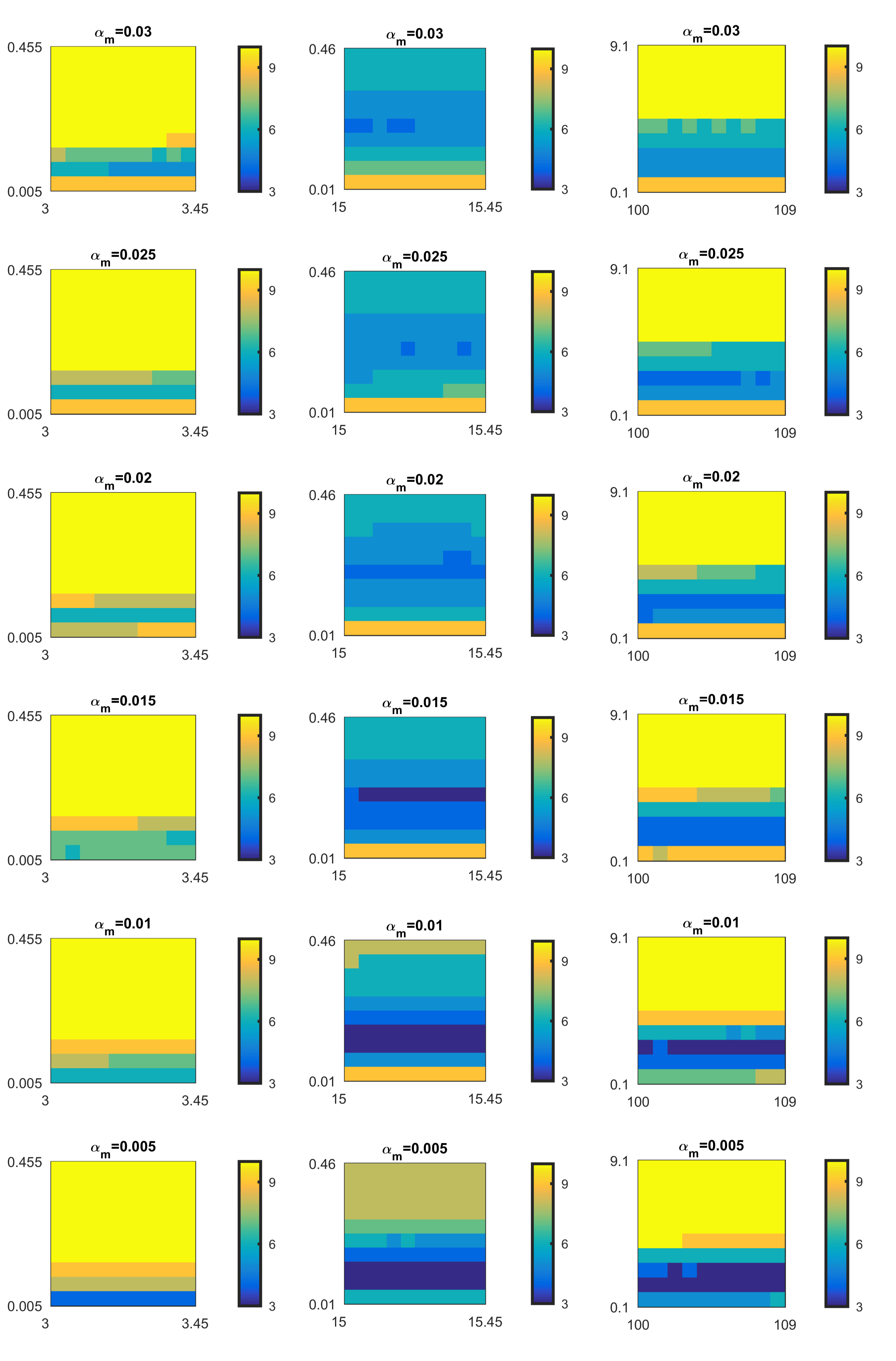

Supplement: S6 Fig — Each column corresponds to one of the three (slow-, intermediate- and fast-switching) regimes. (PNG) [file pcbi.1006000.s010.png]

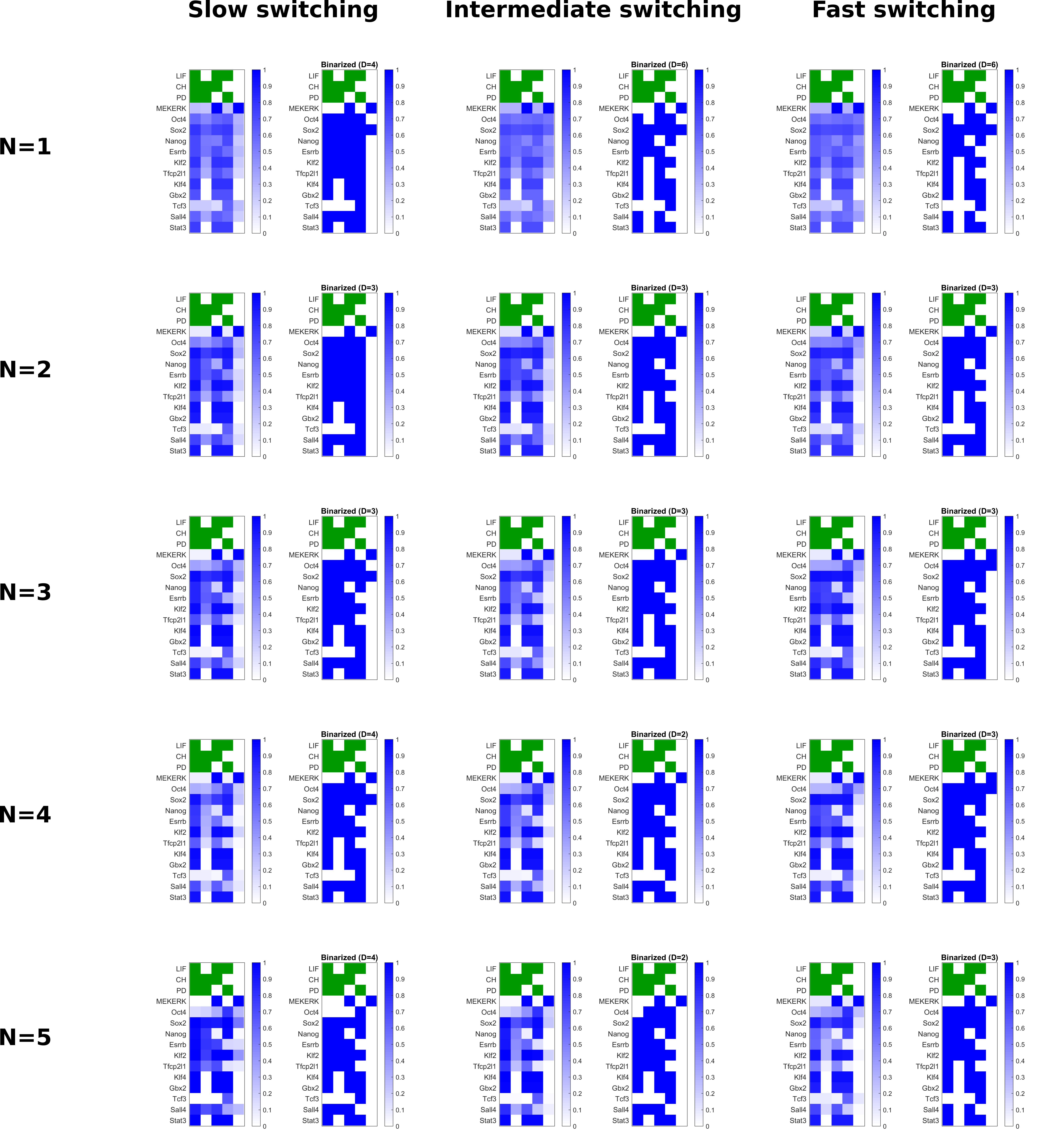

Supplement: S7 Fig — The expression patterns were generated with the parameter set (αm, kon, koff) which has the minimal Hamming distance among the points we sampled in Figs. S2-S7 to the experimental data in Dunn et al. Each column corresponds to one of the three (slow-, intermediate- and fast-switching) regimes. (PNG) [file pcbi.1006000.s011.png]

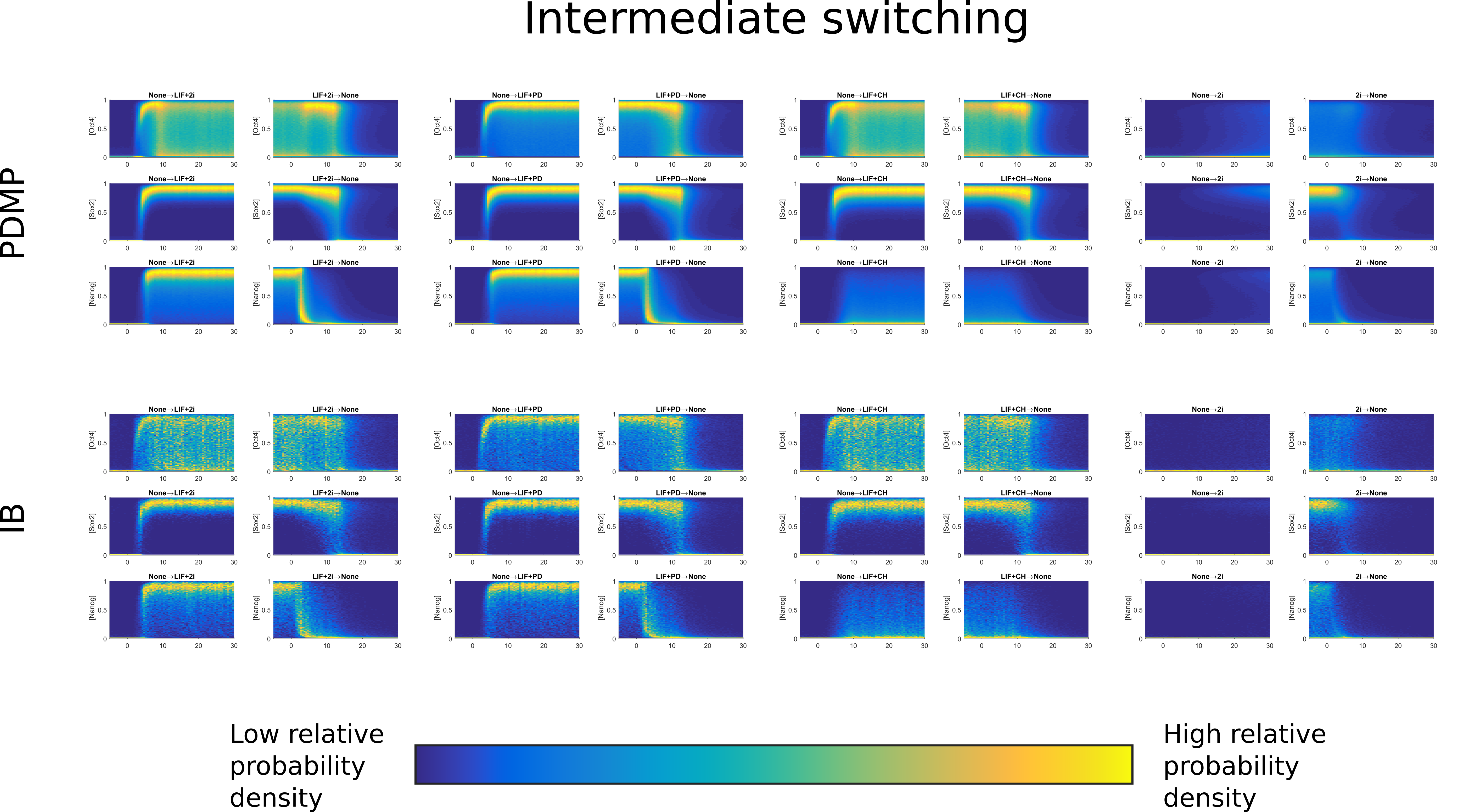

Supplement: S8 Fig — First row: reproduction of Fig 4 of the main text manuscript. Second row: corresponding IB model (with ∼ 100 sample paths) with the same perturbations. (PNG) [file pcbi.1006000.s012.png]
